# Cation Chloride Cotransporter NKCC1 Operates through a Rocking-Bundle Mechanism

**DOI:** 10.1021/jacs.3c10258

**Published:** 2023-12-26

**Authors:** Manuel José Ruiz Munevar, Valerio Rizzi, Corinne Portioli, Pietro Vidossich, Erhu Cao, Michele Parrinello, Laura Cancedda, Marco De Vivo

**Affiliations:** † Laboratory of Molecular Modelling & Drug Discovery, Istituto Italiano di Tecnologia, Via Morego 30, Genoa 16163, Italy; ‡ Biomolecular & Pharmaceutical Modelling Group, Université de Genève, Rue Michel-Servet 1, Geneva CH-1211 4, Switzerland; § Laboratory of Nanotechnology for Precision Medicine, Istituto Italiano di Tecnologia, Via Morego 30, Genoa 16163, Italy; ∥ Laboratory of Atomistic Simulations, Istituto Italiano di Tecnologia, Via Morego 30, Genoa 16163, Italy; ⊥ Laboratory of Brain Development and Disease, Istituto Italiano di Tecnologia, Via Morego 30, Genoa 16163, Italy; # Department of Biochemistry, University of Utah School of Medicine, Salt Lake City, Utah 84112-5650, United States

## Abstract

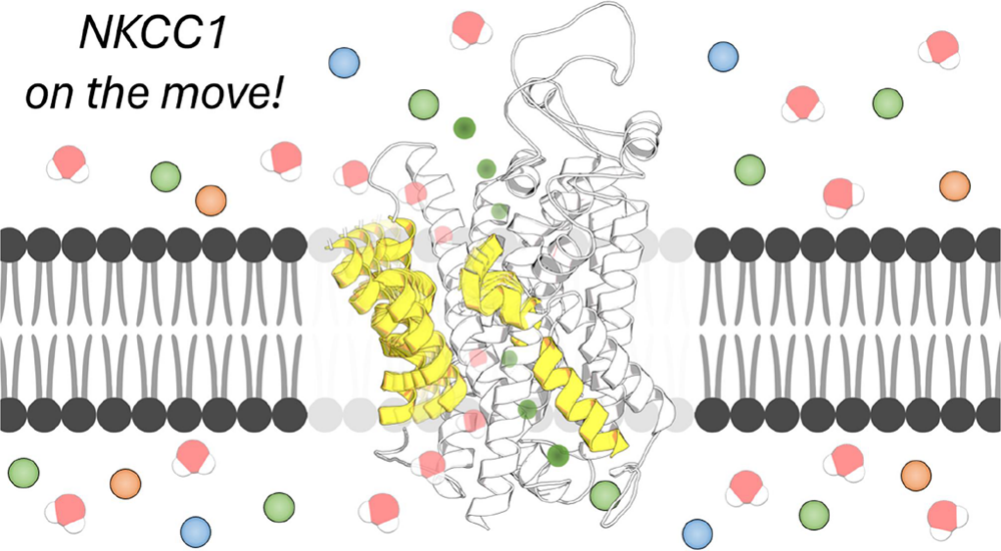

The sodium, potassium, and chloride cotransporter 1 (NKCC1) plays a key role in tightly regulating ion shuttling across cell membranes. Lately, its aberrant expression and function have been linked to numerous neurological disorders and cancers, making it a novel and highly promising pharmacological target for therapeutic interventions. A better understanding of how NKCC1 dynamically operates would therefore have broad implications for ongoing efforts toward its exploitation as a therapeutic target through its modulation. Based on recent structural data on NKCC1, we reveal conformational motions that are key to its function. Using extensive deep-learning-guided atomistic simulations of NKCC1 models embedded into the membrane, we captured complex dynamical transitions between alternate open conformations of the inner and outer vestibules of the cotransporter and demonstrated that NKCC1 has water-permeable states. We found that these previously undefined conformational transitions occur via a rocking-bundle mechanism characterized by the cooperative angular motion of transmembrane helices (TM) 4 and 9, with the contribution of the extracellular tip of TM 10. We found these motions to be critical in modulating ion transportation and in regulating NKCC1’s water transporting capabilities. Specifically, we identified interhelical dynamical contacts between TM 10 and TM 6, which we functionally validated through mutagenesis experiments of 4 new targeted NKCC1 mutants. We conclude showing that those 4 residues are highly conserved in most Na^+^-dependent cation chloride cotransporters (CCCs), which highlights their critical mechanistic implications, opening the way to new strategies for NKCC1’s function modulation and thus to potential drug action on selected CCCs.

## Introduction

The cation chloride cotransporter (CCC) NKCC1 is a modulator of intracellular Cl^–^ concentration in diverse cell types in numerous body organs. Particularly, NKCC1 is expressed in parenchymal brain cells, where it regulates intraneuronal Cl^–^ concentration which in turn is crucial for the modulation of the function of the neurotransmitter GABA, Gamma-aminobutyric acid.^1^ In the majority of cell types, NKCC1 uses an inward-directed Na^+^ gradient to import Na^+^, K^+^, and Cl^–^, in a 1:1:2 stoichiometry.^2^ NKCC1 is also highly expressed on the apical membrane of the choroid plexus, where it plays a major role in producing and regulating cerebrospinal fluid (CSF). Importantly, in recent years, extensive research has shown that an increased intracellular Cl^–^ concentration in neurons is symptomatically related to multiple neuropathologies and also glioblastoma, along with the growing body of literature, showing NKCC1’s important pathological role in increased CSF production.^3−7^ Accordingly, normalization of intracellular Cl^–^ concentration and of CSF hypersecretion in the brain by modulation of CCC (including NKCC1) functions is considered a very promising strategy for neuroscience drug discovery.^3,7−10^ A fundamental understanding of NKCC1’s structure, dynamics, and overall conformational motions for ion passage may therefore open new avenues for therapeutic interventions on many human disorders ranging from brain and hearing to kidney and cancer diseases.

**Figure 1:**
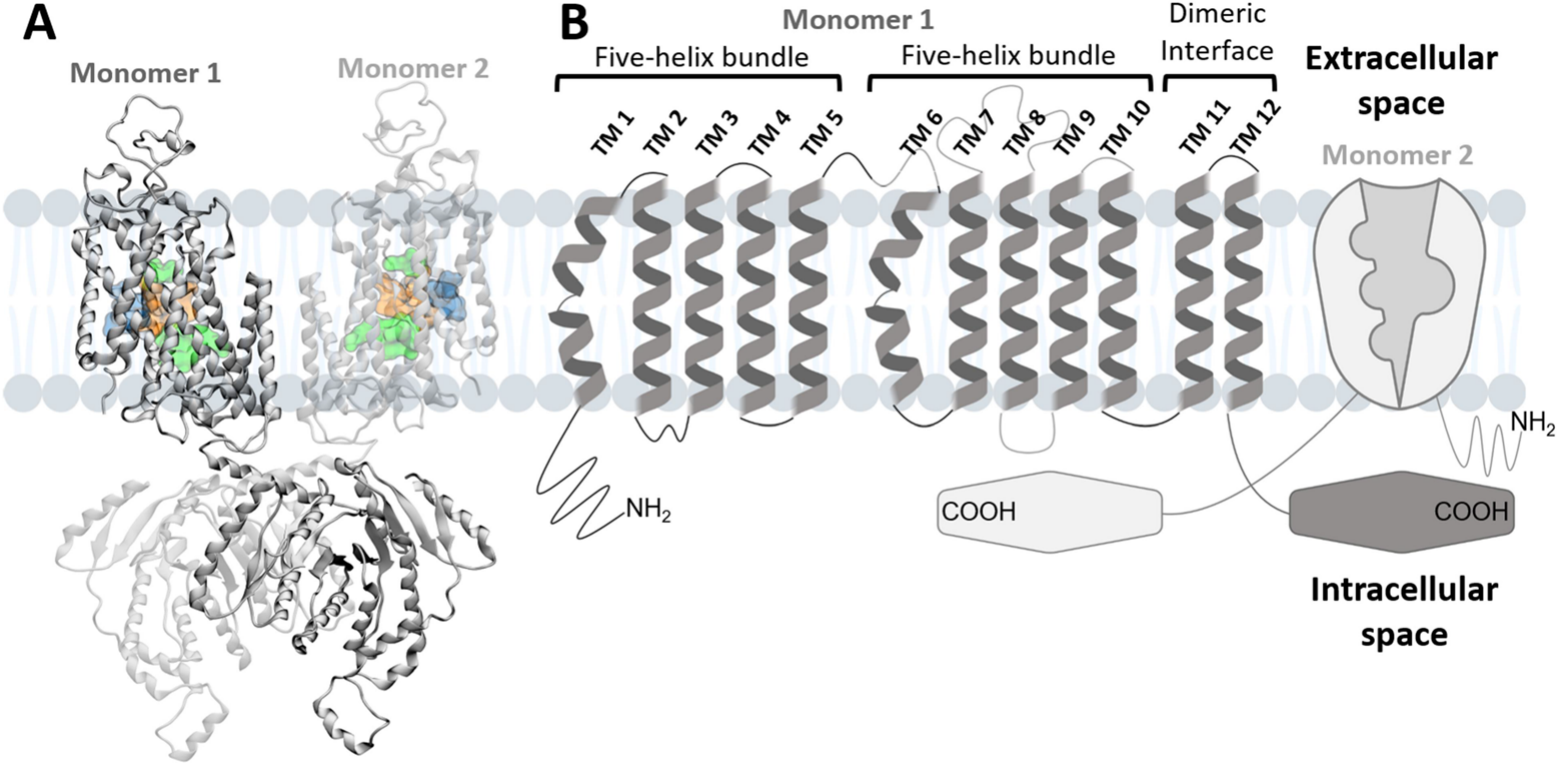
NKCC1’s structure embedded in the membrane. (A) 3D representation of full-length *human* NKCC1 (PDB 7MXO), with each monomer represented in dark and light gray. Each monomer is constructed by three main components: (i) conserved TM domain, composed of 12 helices, which contains all four ion binding sites, shown in green, blue and orange for Cl^–^, Na^+^, and K^+^ ions, respectively; (ii) disordered amino-terminal; and (iii) large carboxy-terminal domains.^13^ All structures of NKCC1, and related CCC’s of the same family,^40^ revealed that the TM helices are organized in two inverted repeats of five-helix bundles—also known as LeuT-fold.^2,16,17,21^ (B) Schematic representation of NKCC1, showing its homodimeric structure with one monomer represented to highlight the transmembrane domains (TMs, dark gray); the other monomer represented to highlight the channel arrangement across the cell membrane (light brown); the two five-helix bundle inverted repeats (TM 1 to TM 5 and TM 6 to TM 10) and the dimeric interface (TM 11 and TM 12). The amino and carboxy-terminal domains are also highlighted.

Interestingly, 15 NKCC1 structures have recently been resolved alone or together with unselective inhibitors (e.g., the FDA-approved diuretic bumetanide). All these recent cryo-EM structures of NKCC1 have clarified several functional features of its multidomain system,^11−15^ revealing a structural similarity to the LeuT-fold transporters.^2,16^ In particular, NKCC1 is a homodimer, where each monomer is constructed by three main components: a conserved transmembrane (TM) domain composed of 12 helices organized in two inverted repeats of five-helix bundles, which contain all four ion binding sites (Figure 1A). Two additional intracellular domains are the disordered amino-terminal and the large carboxy-terminal domains, which are related to NKCC1 activation (Figure 1B).^13^

**Figure 2:**
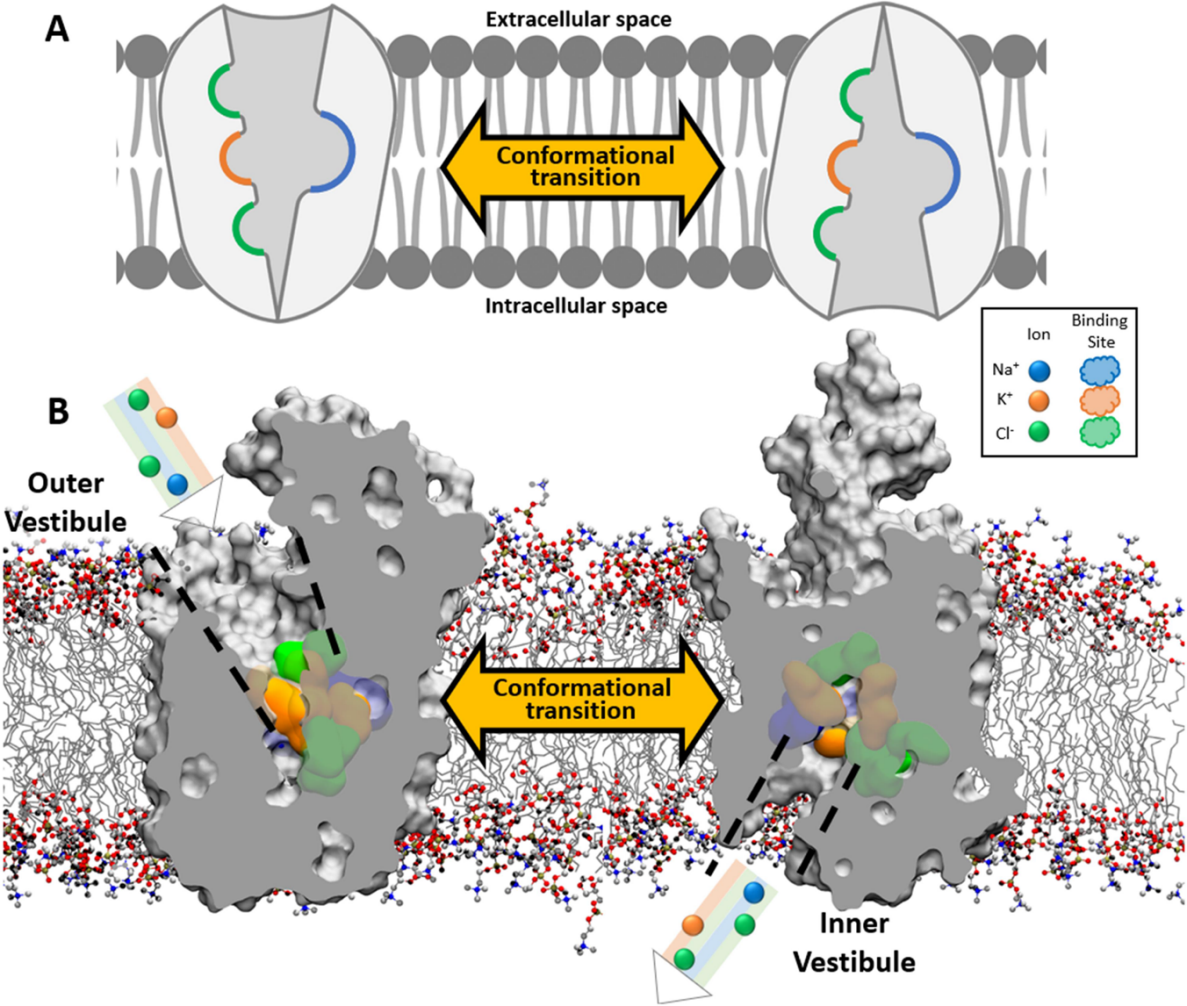
Essential conformational transition for alternating accessibility of ion binding sites allows NKCC1 ion transport. (A) Schematic representation of outward open (left) and inward open (right) NKCC1 conformation. Ionic binding sites are exposed to the extra and intracellular side of the membrane, respectively. (B) In gray, atomic surface representation of outward open (left) and inward open (right) NKCC1 conformation. Ionic binding sites for Na^+^, K^+^, and Cl^–^ are shown as colored surfaces (blue, orange, and green, respectively), and ions are represented as colored spheres. Outer (left) and inner (right) vestibules, open to the extra and intracellular sides of the membrane, are highlighted with discontinuous black lines.

The NKCC1 structures recently resolved have also confirmed that NKCC1 operates transiting from conformations where the ion binding sites are exposed either to the extracellular or to the intracellular side of the membrane.^17^ That is, NKCC1 must transit dynamically through two distinct conformational states: an outward open (OO) state, in which the transporter binds to ions from outside of the cell, and an inward open (IO) state, in which the protein releases the ions to the inside of the cell (Figure 2). However, the mechanism for the IO ↔ OO conformational transitions in NKCC1 is unclear.

In this context, we have explored here different molecular mechanisms for IO ↔ OO conformational transitions in NKCC1 and ion transport in NKCC1 embedded in the membrane. To do so, we have used extensive classical equilibrium molecular dynamics (MD) simulations and deep-learning-guided enhanced sampling free energy calculations.^18,19^ We also validated our in silico evidence by biological functional studies in cell cultures with targeted NKCC1 mutagenesis. Combining our molecular modeling and molecular biology experiments, we clarified the complex IO ↔ OO conformational transition in NKCC1 and revealed that it operates through a rocking-bundle mechanism. In addition, we have identified specific interhelical dynamical contacts that we have found to be fundamentally involved in the NKCC1’s transport cycle, favoring water diffusion through the transporter. Based on structural similarities and biochemical data analyses, we propose that our findings could be extended to all Na^+^-dependent CCCs.

## Results

### NKCC1 Operates via a Rocking-Bundle Mechanism for Conformational Transitions

To investigate the mechanism of the NKCC1 ion transporter, we ran extensive classical MD simulations coupled to the on-the-fly probability enhanced sampling (OPES) method to handle elaborated deep-learning-derived collective variables (CVs) needed to capture complex dynamical phenomena^19,20^ and accelerate a meaningful sampling of the underlying conformational space. Notably, these simulations of atomistic models embedded into the membrane were grounded on recently resolved human NKCC1 structures. Specifically, our realistic models were based on the structure of the IO state^13^ and a partially loaded OO state of NKCC1 (Figure 2B).^15^ We initially focused on NKCC1 model systems in which ions bound at the vestibules are absent (i.e., the ions’ external gates at the transporter, see Figure 2). This setup allowed us to focus our exploration on the mechanistic transitions between the IO ↔ OO NKCC1 conformations. As a result, we could sample 16 IO ↔ OO conformational transitions (Figure S4) and collected over ∼2 μs of trajectories from a total of 3 independent enhanced sampling simulations, in addition to ∼2.5 μs of equilibrium MD runs.

**Figure 3:**
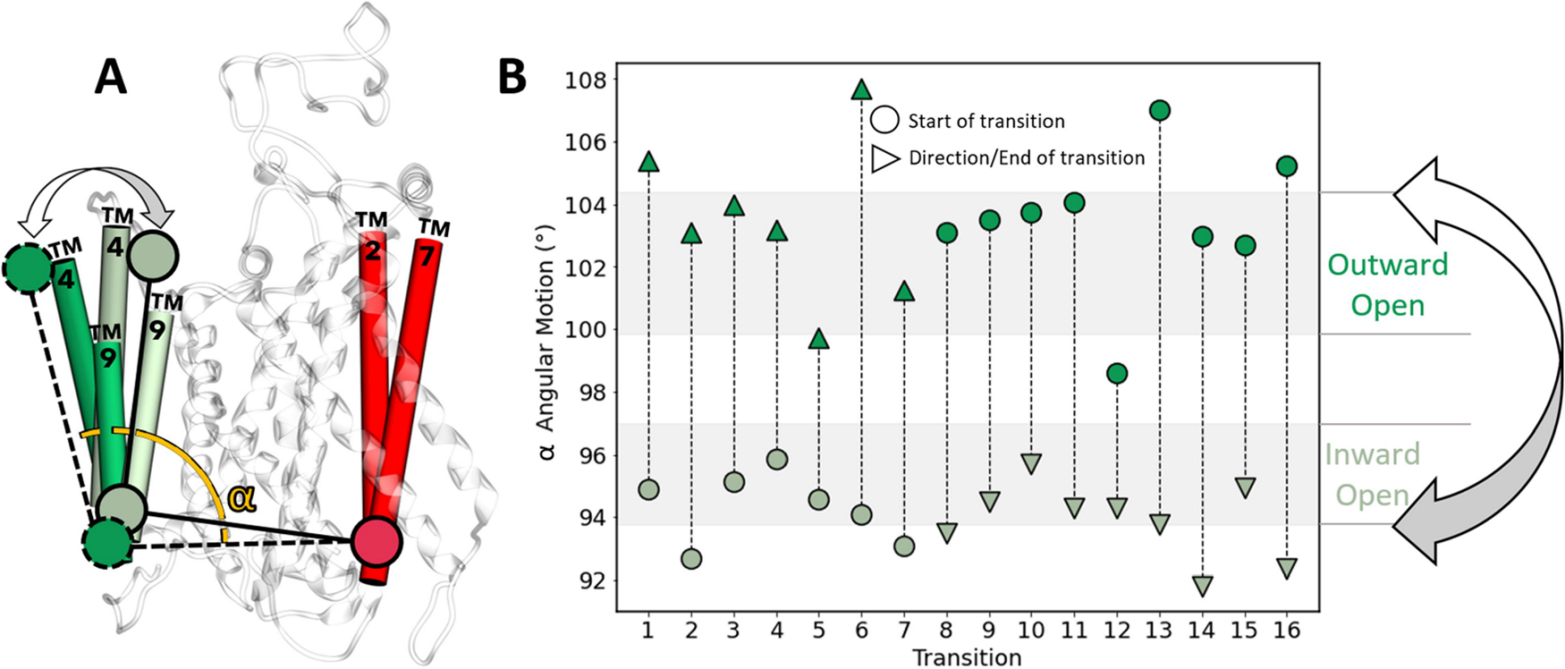
Rocking-bundle angular motion of specific NKCC1 TMs facilitates alternate accessibility of ion binding sites. (A) Schematic representation of NKCC1’s angular motion, defined as the change of the angle (α) between TM 4 and TM 9 (in green) and TM 2 and TM 7 (in red), during the conformational transition between the outward open state (bright green) and inward open state (dim green), calculated from the centers of mass of the backbone atoms from the extracellular and intracellular tip of TM 4 and TM 9 and the intracellular tip of TM 2 and TM 7 (in red). (B) Quantification of TM 4 and TM 9 angular motion represented by the angle α through 16 conformational transitions from OPES Explore simulations. The light brown horizontal bars represent the outward open and inward open average angle α ± 1SD calculated from 1 μs of equilibrium molecular dynamics (MD). Circles represent the starting point of each transition, whereas the triangles represent the end point of the same transition and its direction. Circles and triangles are colored depending on the NKCC1 conformation they represent (bright green for outward open and dim green for inward open).

There are diverse alternating access mechanisms that could allow NKCC1 to shuttle ions inside the cell, passing across the cell membrane.^17,21^ For example, the “rocking-bundle” or the “elevator” mechanism are equally plausible alternating mechanisms for ions transport across the membrane. Significantly, these distinctive alternating mechanisms differ in the active motions of specific TM helices, which must assist the dynamic passage of ions. The IO ↔ OO conformational transitions observed in our simulations revealed that NKCC1 operates through the “rocking-bundle mechanism”.^22^ In particular, our computational evidence shows the exact protein motions for NKCC1’s function, clarifying which specific dynamics operates within the general ‘alternating access mechanism’. We found indeed that the inner and outer vestibules of NKCC1 alternate their accessibility using the rocking-bundle mechanism for NKCC1 characterized by the motion of TM helices TM 4 and TM 9. In fact, both TM 4 and TM 9 stably maintained their initial structure for over ∼1 μs in both IO and OO states during our equilibrium MD, with an average root-mean-square deviation (RMSD) of 0.68 ± 0.14 and 0.72 ± 0.11 Å, respectively. However, in all the 16 IO ↔ OO transitions (Figure 3B), these TM helices showed a concerted angular motion, with a rotation of at most 14° with respect to the intracellular extreme of TM 2 and TM 7 - both located within the *static* domain formed by TM 1, TM 2, TM 6 and TM 7 of NKCC1 (Figure 3A), which is used for structure alignment. The RMSD of TM 4 and TM 9, in the IO and OO states of the cryo-EM structures, was 4.21 Å (vs = 1.75 Å for the whole remaining TMs). This confirmed the concerted movement of these two TM helices to be in line with an angular motion typical of the rocking-bundle mechanism (Figure S1;^23^).

**Figure 4:**
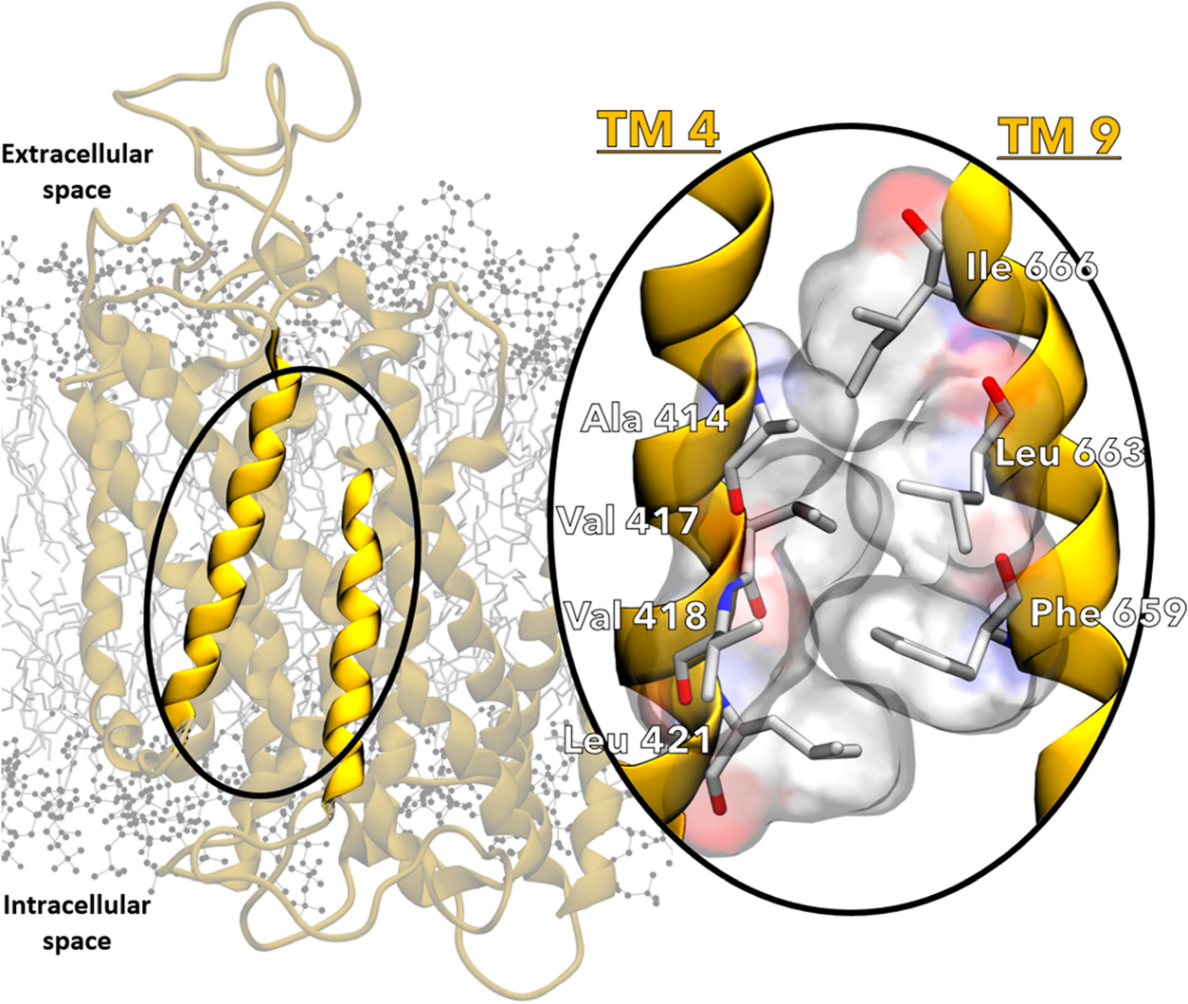
Stabilization of the hydrophobic interface between TM 4 and TM 9 allows for their cooperative action. Representation of *human* NKCC1 embedded in the cell membrane, with TM 4 and TM 9 highlighted in bright yellow. Inset on the right: Higher magnification of the hydrophobic interface between TM 4 and TM 9 (highlighted by the oval), which allows for their cooperative angular motion. Relevant residues are shown as sticks with their atomic surfaces pictured in red (oxygen), blue (nitrogen), gray (carbon), and white (hydrogen).

**Figure 5:**
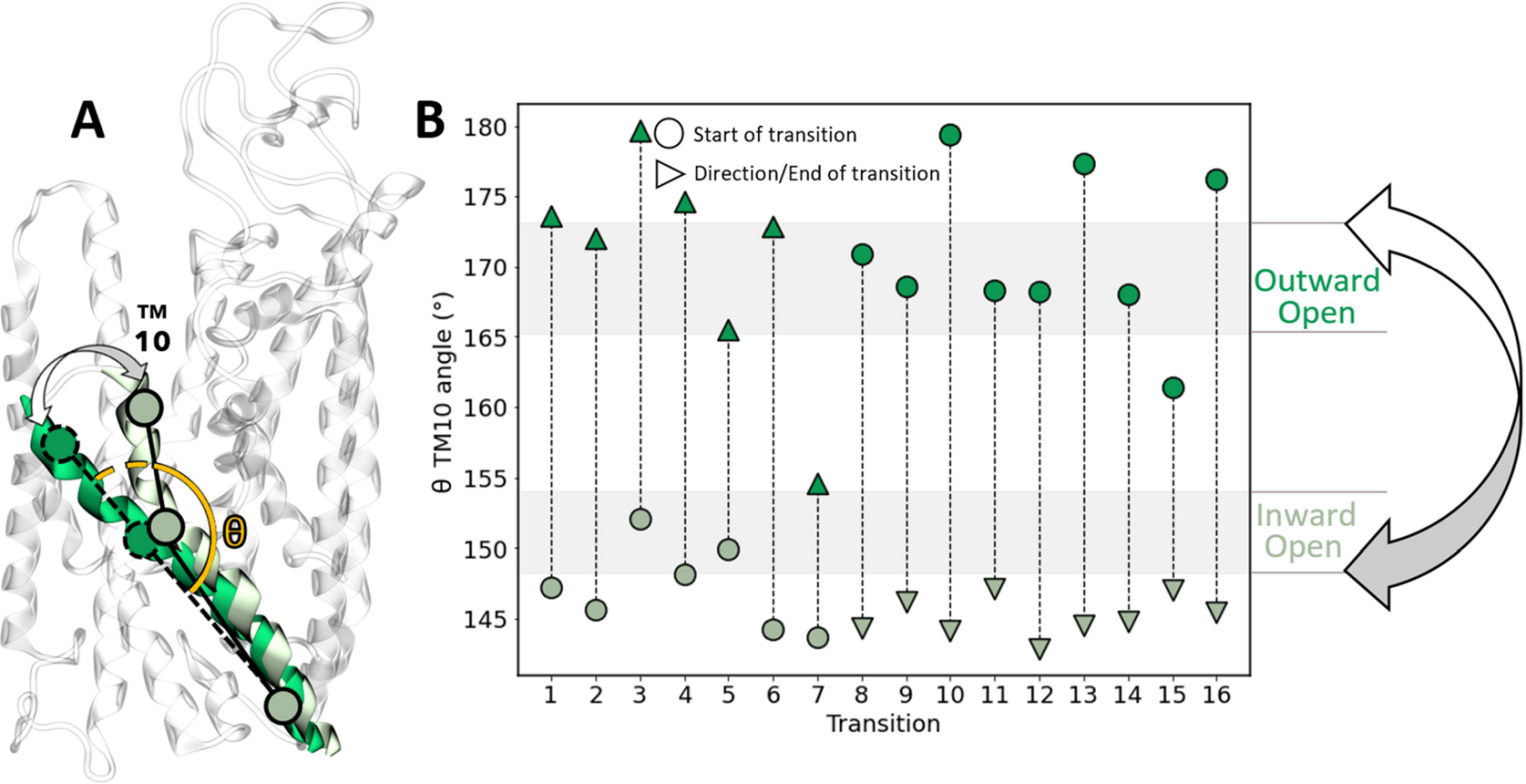
NKCC1 TM 10's corking motion modulates ion/water access to the outer vestibule. (A) Schematic representation of NKCC1 TM 10's corking motion, defined as the change of TM 10's (in green) intrahelical angle (θ), during the conformational transition between the outward open state (bright green) and inward open state (dim green), calculated from the centers of mass of the backbone atoms from TM 10’s intra and extracellular tips, and the backbone atoms where TM 10 bends. (B) Quantification of TM 10's corking motion represented by the angle θ through 16 conformational transitions from OPES Explore simulations. The light brown horizontal bars represent the outward open and inward open average angle θ ± 1SD calculated from 1 μs of equilibrium molecular dynamics (MD). Circles represent the starting point of each transition, whereas the triangles represent the end point of the same transition and its direction. Circles and triangles are colored depending on the NKCC1 conformation they represent (bright green for outward open and dim green for inward open).

**Figure 6:**
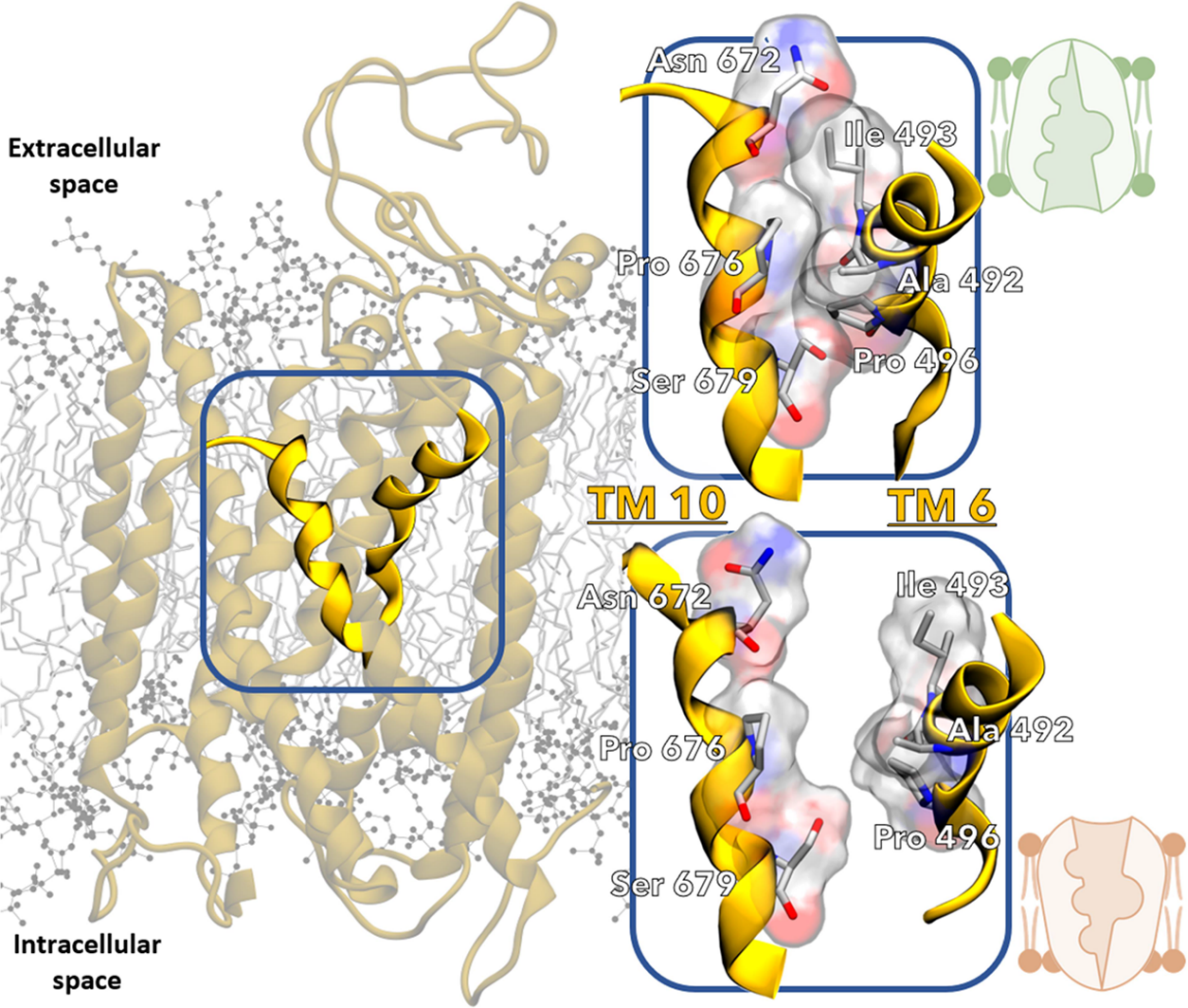
Interface between TM 10 and TM 6 highlights crucial interactions that determine accessibility of extracellular binding sites. Representation of *human* NKCC1 embedded in the cell membrane, with TM 10 and TM 6 highlighted in bright yellow. Inset on the top right: In the IO state, as shown by the light green schematic representation, interacting residues at the TM 10 and TM 6 interface are shown as sticks with their atomic surfaces (pictured in red—oxygen, blue—nitrogen, gray—carbon, and white—hydrogen), blocking solvent access to the outer vestibule. Inset on the bottom right: In the OO state, as shown by the light orange schematic representation, previously interacting residues at the TM 10 and TM 6 interface are now shown to be too far apart to form bonds.

Interestingly, we found that TM 4 and TM 9 operate as a joint structural motif due to several hydrophobic interactions at their interface, in agreement with previous structural observations.^15^ Here, we found that these hydrophobic interactions involved Ala 414, Val 417, Val 418 and Leu 421 from TM 4, while Phe 659, Leu 663 and Ile 666 from TM 9 (Figure 4). These interactions, statically present also in the cryo-EM structures,^11−15^ were stably maintained during our equilibrium MD simulations, in both the IO and OO states. In addition, we observed the crucial involvement of TM 10, which was key to the NKCC1’s rocking-bundle mechanism in our simulations. TM 10 is connected by a short loop on the extracellular side to TM 9. This connection made TM 10 susceptible to conformational changes due to TM 4 and TM 9’s angular motion, when transiting from the IO to the OO state (and *vice versa*). In our simulations, this motion modulated the solvent accessibility to the outer vestibule of NKCC1. In detail, TM 10 rested at an angle of 151.2 ± 2.9° during our equilibrium MD of the IO state, while it conserved an angle of 169.2 ± 4.0° in the OO state throughout the equilibrium MD. However, during IO → OO transitions, TM 4 and TM 9’s angular motion was critical to drag the TM 10's extracellular tip outward, allowing the solvent to access the outer vestibule (Figure 5A). In the IO → OO transitions, TM 10 was dynamically straightened (again, from 151.2 ± 2.9 to 169.2 ± 4.0°). This broke the interatomic contacts at the TM 10-TM 6 interface (i.e., Asn 672 with Ile 493, Pro 676 with Ala 492 and Ser 679 with Pro 496, at the TM 10 and TM 6, respectively; Figure 6, inset on the top right). Notably, Pro 496 and Ser 679 interacted with water molecules, thus potentially assisting the flooding of water into the NKCC1’s outer vestibule. This mechanism is again in line with a rocking-bundle mechanism. In addition, in agreement with this evidence, during OO → IO transitions TM 4 and TM 9’s angular motion pushed TM 10's extracellular tip inward, therefore disabling solvent accessibility to the outer vestibule of NKCC1. Taken together, these results show that the inward ↔ outward motion of TM 10 modulates NKCC1 solvent accessibility, as observed in all 16 conformational transitions (Figure 5B).

Next, we also investigated the possibility for NKCC1 to operate by the so-called “elevator mechanism”, which would be an alternative to the rocking-bundle one. The possibility of identifying an elevator mechanism was evaluated by measuring the vertical translation of TM 4 and TM 9 vs TM 2 and TM 7, which are part of the *static* domain. We found that TM 4 and TM 9 did not show any vertical translation during any of the conformational transitions, as their relative positions in the IO and OO state cryo-EM structures were not vertically translated. This is not what would be expected for an elevator mechanism (Figure S5). Altogether, our results indicate a rocking-bundle mechanism for NKCC1 function while excluding an elevator mechanism.

**Figure 7:**
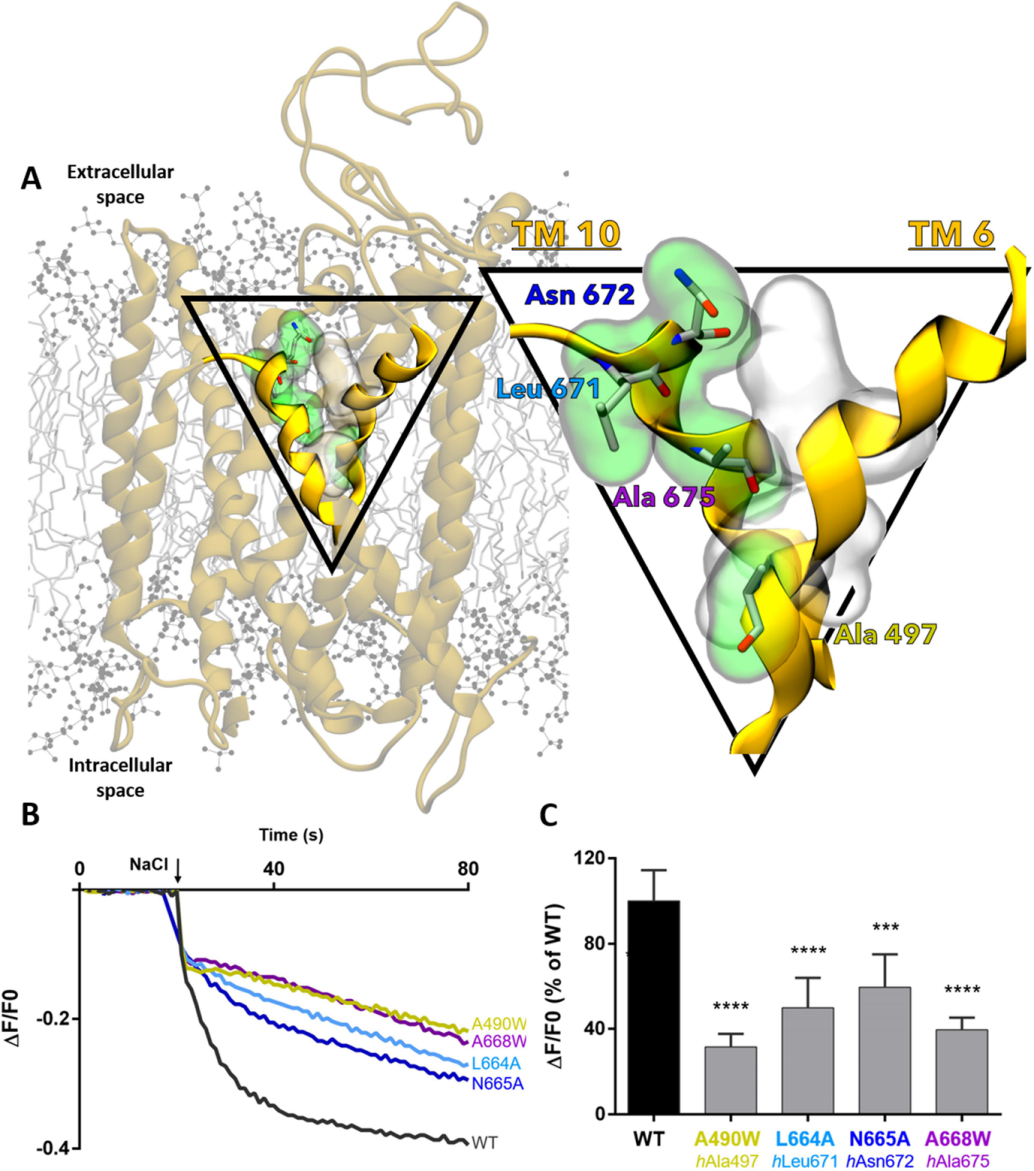
Mutagenesis targeting residues from TM 10 and TM 6 highlight their functional relevance. (A) Representation of *human* NKCC1 embedded in the cell membrane, with TM 10 and TM 6 highlighted in bright yellow. Inset on the right: The interface between TM 10 and TM 6 where homologous mutated residues are shown as sticks and are highlighted in green surface. Namely, these residues are mouse A490W (*human* Ala 497), mouse L664A (*human* Leu 671), mouse N665A (*human* Asn 672), and mouse A668W (*human* Ala 675). Gray surface represents the position of residues that mainly form/break interactions throughout TM 10's corking motion. (B) Example traces obtained in the Cl^–^ influx assay on HEK293 cells transfected with the WT NKCC1 transporter or NKCC1 mutated at different residues. The arrow indicates the addition of NaCl (74 mM) to initiate the NKCC1-mediated Cl^–^ influx. (C) Quantification of the mouse NKCC1 inhibitory activity using the Cl^–^ influx fluorescence assay in HEK293 cells. A fluorescence signal decrease, corresponding to a decrease in NKCC1 transporter activity, was observed for all the cells transfected with NKCC1 mutants. Data are normalized and the average of the last 10 s of kinetics is plotted (Δ*F*/*F*0). Data are presented as a percentage of the WT. Data represent mean ± SEM from 3 to 4 independent experiments (Kruskal–Wallis one way ANOVA, H = 216, DF = 6, followed by Dunn’s post hoc test on multiple comparisons, *** *P* = 0.0002, **** *P* < 0.0001).

To test the functional importance of TM 10's motion and association with TM 6 as evidenced by our simulation data, we performed cell and molecular biology experiments in standard stable cell lines (HEK293 kidney cells) transfected with 4 diverse mutants (Ala490Trp, Leu664Ala, Asn665Ala and Ala668Trp) of mouse NKCC1. Specifically, the NKCC1 constructs were generated with 4 mutations on residues located at the interface between TM 10 and TM 6. These TM residues, all located at the outer vestibule (Figure 7A), were selected because of their involvement in the dynamic interaction network highlighted by our MD simulations for NKCC1 conformational transitions. Next, we performed in vitro Cl^–^ influx assay^24−26^ measurements on HEK293 cells transfected with one of the four *mouse* NKCC1 mutants, at the time.

In our in vitro cellular assay, all mutations lead to a decrease in ion transport function, when compared to the wild-type (WT) *mouse* NKCC1 (Figure 7C). In detail, the selected mutations were Ala490Trp (equivalent to *human* NKCC1 Ala497Trp at TM 6, alignment shown in Figure S9). This mutation introduces a bulky side chain that disrupts the formation of the Ser 679 – Pro 496 interaction at the TM 6 – TM 10 interface at the outer vestibule. Ala490Trp mutation led to reduced transport by a factor of 1.7 when compared to the WT NKCC1. We infer that by affecting TM 10's motion, the NKCC1’s IO state is destabilized, therefore disrupting ion transport. Additionally, the mutations Leu664Ala, Asn665Ala and Ala668Trp (equivalent to *human* Leu671Ala, Asn672Ala and Ala675Trp, see Figure S9 - all residues located in the extracellular tip of TM 10) also led to a reduction in ion transport by a factor of 1.4, 1.3, and 1.6, respectively, when compared to the wild-type NKCC1 (Figure 7C). In this context, the Leu664Ala and Asn665Ala mutations on TM 10 remove the side chain that interacts with the Asn672 and Ile493 residues on TM 10 and TM 6, respectively. The mutation Ala675Trp introduces a bulky side chain that disrupts the formation of the Pro 676 – Ala 492 interaction, weakening the TM 10 mobility (Figure 7A).

Overall, our simulations and mutagenesis data support a rocking-bundle mechanism for NKCC1 conformational transitions, which critically relies on the TM 4 and TM 9’s angular motion and dynamic contacts of residues at the TM 6 – TM 10 interface, with TM 10 that seems to also modulate the access of the solvent to the outer vestibule.

### Free Energy Simulations Characterize the Previously Elusive NKCC1’s Occluded State

**Figure 8:**
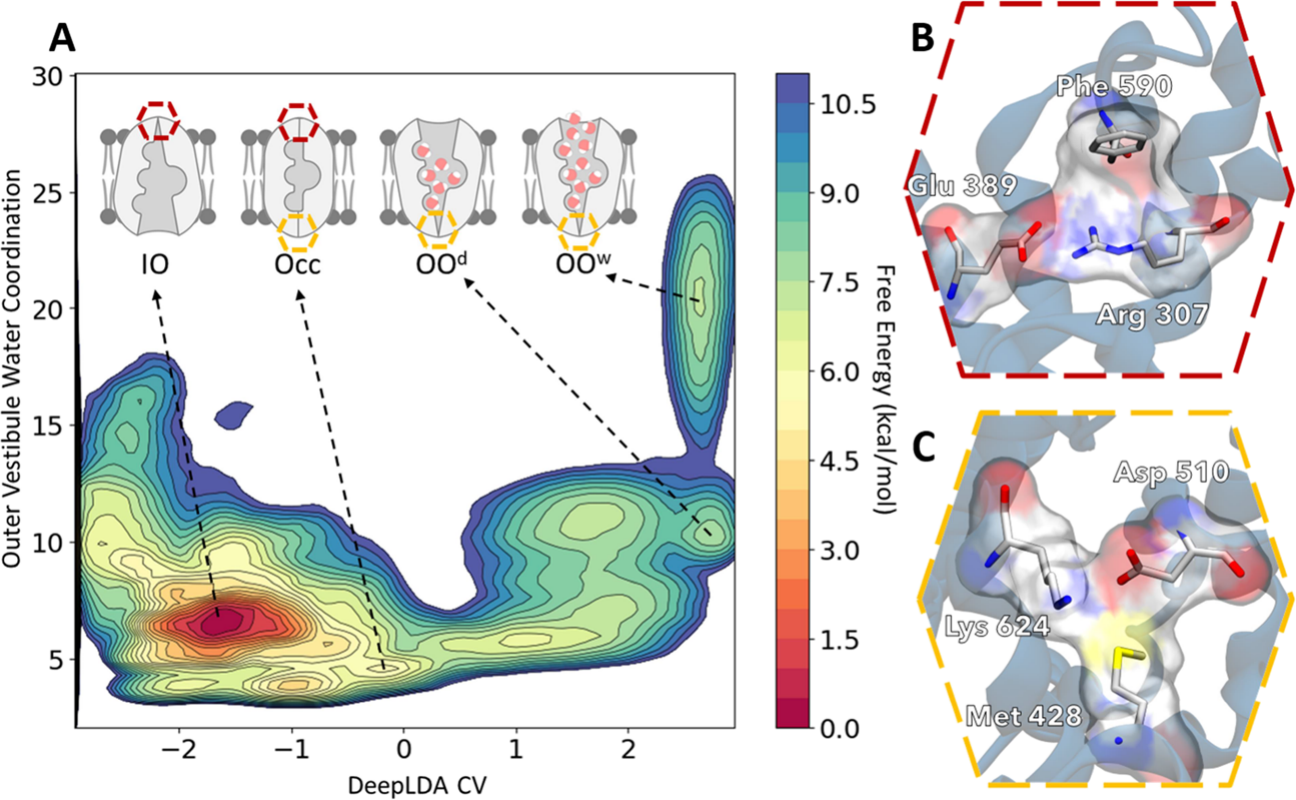
Free energy surface identifies relevant NKCC1 conformations for the inward open ↔ outward open transition. (A) Representation of the Free Energy Surface of the conformational transition between *human* NKCC1 IO and OO states computed by OPES Explore over the DeepLDA collective variable and the outer vestibule water coordination collective variable. Energetical basins are highlighted with a schematic representation of the conformation they identify. These are, namely: the IO state, the occluded state (labeled Occ), the OO^d^ state (outward open “dry” – lower outer vestibule hydration) and OO^w^ (outward open “wet” – higher outer vestibule hydration. (B) Higher magnification of the hexagon in A representing the main gating interactions that occlude the outer vestibule and block solvent access to the ionic binding sites. Relevant residues are shown as sticks with their atomic surfaces pictured in red (oxygen), blue (nitrogen), gray (carbon) and white (hydrogen). (C) Higher magnification of the hexagon in A representing of the main gating interactions that occlude the inner vestibule and block solvent access to the ionic binding sites. Relevant residues are shown as sticks with their atomic surfaces pictured in red (oxygen), blue (nitrogen), yellow (sulfur), gray (carbon) and white (hydrogen).

Then, we analyzed the free energy surface (FES) for IO ↔ OO conformational transitions of NKCC1 as revealed by the OPES Explore algorithm and CVs used to enhance the sampling of the complex dynamical transitions between open conformations of the inner and outer vestibules of the transporter (see the paragraph above and Methods section). Our simulations revealed 4 distinct free energy minima that correspond to conformational states of NKCC1. Notably, the IO state is located in the deepest minimum. We found that the IO state basin was centered on a value of −1.6 in the CV dimension (Figures 8 and S6). Further equilibrium MD starting from snapshots extracted from this basin showed a stable IO model (Figure S7A). On the other hand, the OO state was distributed into a shallow area located at ∼2.6 in the CV dimension of the FES. However, we found this area to cover different hydration states of the transporter (OO^w^ and OO^d^ in Figure 8). That is, both OO^w^ and OO^d^ are stable OO state conformations that differ in the extent of their water hydration (Figure S7B). Indeed, the outer vestibule in the OO^d^ minimum has a coordination number of waters of ∼10.5, while the outer vestibule in OO^w^ showed a coordination number of ∼20.5.

Importantly, our exploration of FES also captured NKCC1 in its occluded state (Occ, Figure 8A). Notably, such NKCC1 occluded state had been only hypothesized to exist,^27^ as it has never been structurally determined, likely due to its transitory nature. This state was located at ∼0 in the CV space with an outer vestibule hydration coordination number of 4.8. Notably, such an occluded state emerged naturally from our deep-learning-guided enhanced sampling simulations trained solely on the IO and OO state conformations.

**Figure 9:**
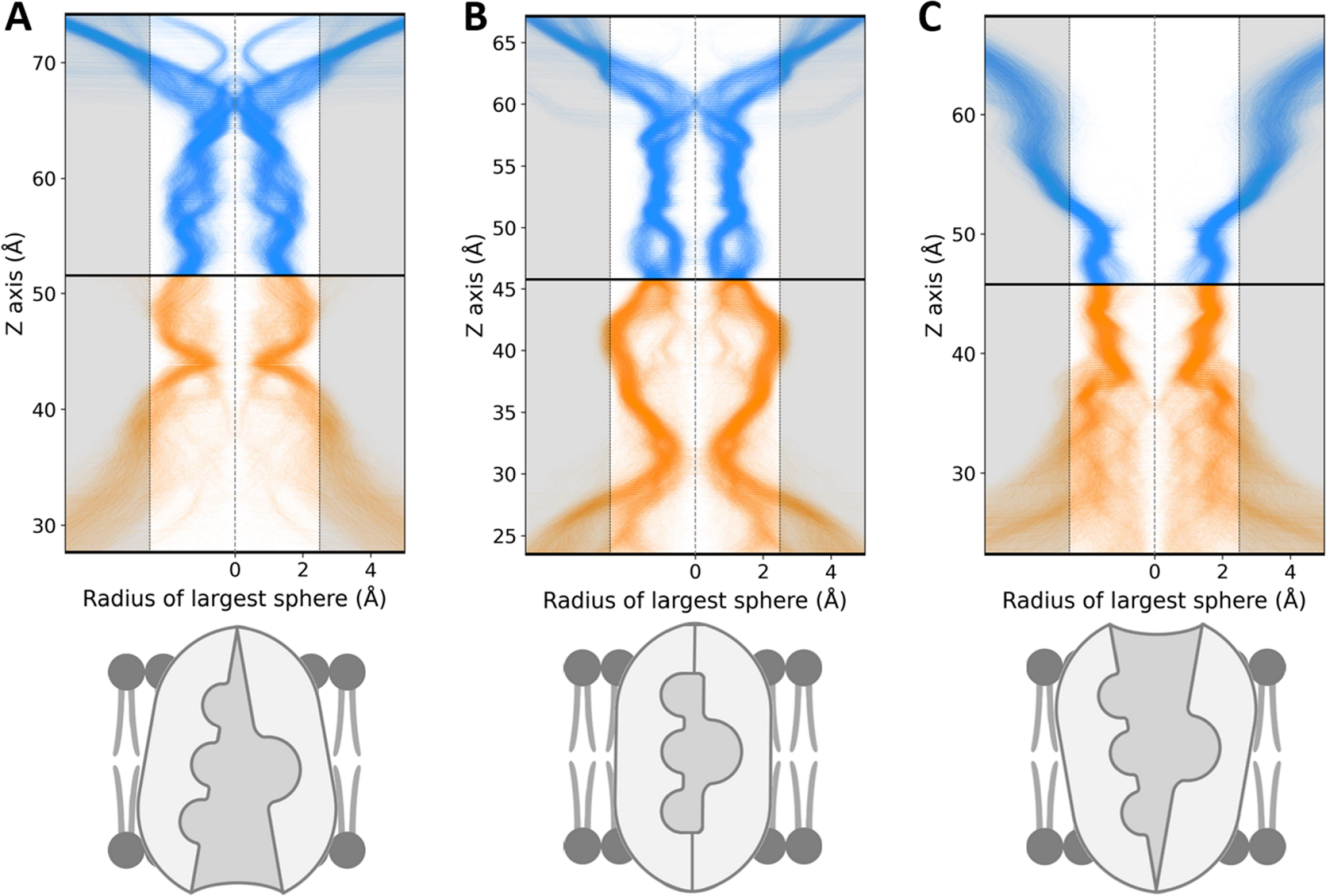
Pore profile confirms distinct binding-site accessibility of NKCC1 states along the inward open ↔ outward open transition. (A–C) Pore profile of the IO state (A), the occluded state (B), and OO state (C), schematically represented at the bottom. These profiles were obtained by calculating the radius of the largest sphere along the *Z*-axis of the ion translocation cavity, and then plotting the pore profile of several snapshots from their corresponding equilibrium MD simulations, computed by the software HOLE. Pore profiles were mirrored around radius 0 for visual clarity. The blue lines represent the outer vestibule, and the orange lines represent the inner vestibule of NKCC1.

In our simulations, the occluded state depicts NKCC1 with its ion binding sites inaccessible to the solvent, from either side of the membrane (Occ in Figure 8A), and it is characterized by having both outer and inner vestibules occluded. The outer vestibule has an extensive intermolecular interaction hub. This was built up by Arg 307, which formed a salt bridge with Glu 389, and cation-π interactions with Phe 590 (Figure 8B). Additionally, the interactions between Asn 672-Ile 493, Pro 676-Ala 492 and Ser 679-Pro 496 were fully formed in the NKCC1 occluded state, thus occluding access of the extracellular solvent to the outer vestibule. On the other side, the inner vestibule was occluded to the intracellular solvent due to several other interactions among residues. Those interactions were mostly conserved also in the OO state of the cryo-EM structure.^15^ Primarily, these interface residues’ interactions are formed after the shifting of Met 428 and the consequent occlusion by intracellular loop 1. Interestingly, the salt bridge between Asp 510 and Lys 624, formed in the OO state where it occludes the inner vestibule (Figure 8C), is not formed in the occluded state. Notably, the occluded state was maintained for over 100 ns in our equilibrium MD simulations, which started from configurations of the Occ state visited during the enhanced sampling trajectories (Figure S7C), with an RMSD of 1.00 ± 0.11 Å. In these simulations, all ion binding sites remained inaccessible to the solvent from either side of the membrane, as shown in the occluded basin pore profile in Figure 9B. Notably, this differs greatly from the IO and OO state equilibrium MD pore profiles (Figure 9A,C, respectively), confirming that such a transitory state is structurally distinct from the IO and OO states. Ultimately, the occluded state is therefore the only transitory conformation that ensures solvent inaccessibility to the ion binding sites from both the intra and extracellular vestibules.

In our FES (Figure 8A), the IO state transited to the occluded state, with a barrier of ∼5.5 kcal/mol. From this transitory occluded state, there is another barrier of ∼5.5 kcal/mol to reach the OO^w^ state. Interestingly, the reverse transition appears less energetically costly, as the OO^w^ state transited to the occluded state with a barrier of ∼3.5 kcal/mol and then followed by a ∼0.5 kcal/mol barrier to reach the IO state. Therefore, the IO state was ∼7 kcal/mol more stable than the OO^w^ state. Taken together, our simulations depict IO ↔ OO transitions passing though the occluded state, further corroborating the rocking-bundle mechanism for NKCC1 function.

Then, we determined the free energy for ion binding to the NKCC1 OO state. According to previously published kinetic data,^28^ ions bind to NKCC1 from the extracellular side of the membrane in the order Na^+^, Cl^–^, K^+^, Cl^–^. We first simulated Na^+^ binding to the unloaded OO state (Figure S11A), and found a Δ*G*_bind_ ≈ −7.5 kcal/mol, with a binding barrier of ∼5.0 kcal/mol. We then evaluated binding of the first Cl^–^ ion to a Na^+^-bound OO state (Figure S11B), which returned a Δ*G*_bind_ ≈ 1.0 kcal/mol, with an energy barrier of ∼4.0 kcal/mol. Next, we evaluated K^+^ binding to a Cl^–^-Na^+^-bound OO state (Figure S11C), and we obtained a Δ*G*_bind_ ≈ of 1.0 kcal/mol, with a barrier of ∼4.5 kcal/mol. After these ion binding simulations, we ran additional ∼100 ns of equilibrium MD of each system and observed that all these partially/fully loaded states were stable, i.e., all ions remained stably coordinated to their binding site throughout the simulations time. Interestingly, within the first 5 ns of the K^+^-Cl^–^-Na^+^-bound OO NKCC1 state, we observed the spontaneous binding of the second Cl^–^ ion to its binding site, leading to the fully loaded OO state of NKCC1, which was stably maintained for over 1 μs.

We analyzed the ion binding sites and ion coordination to inspect possible effects of the conformational transition OO → IO on those ion binding sites. First, we observed that ion loading does not affect the overall structure of NKCC1 (RMSD between the loaded and unloaded states: IO = 1.71 Å, occluded = 1.97 Å, OO = 1.63 Å) and that the fully loaded occluded → IO state conformational transition is characterized by the same angular motion of TM 4 and TM 9 (Figure S10). In addition, we noticed that all ions remained bound to their respective sites throughout the conformational transition OO → IO, although minor changes were detected. In detail, the Na^+^ binding site is composed by residues: Leu 297, Trp 300, Ala 610, Ser 613, and Ser 614. Na^+^ maintains its interactions with most of its coordination sphere in the fully loaded OO, occluded and IO states (Figure S12A). The only state-dependent interactions are between Na^+^ and Ala 610. This distance started at an average length of 2.7 ± 0.7 Å in the OO state (i.e., tightly bound) and increased in both occluded and IO states to 4.4 ± 0.5 and 4.6 ± 0.4 Å, respectively (i.e., loosely bound). The interactions of the first Cl^–^ to its binding site (closest to the intracellular side), are with residues: Gly 500, Ile 501, Leu 502, and Tyr 686. These remained unchanged between the fully loaded OO, occluded and IO states (Figure S12B). Most of K^+^ interactions with its binding site, which is composed by residues: Asn 298, Ile 299, Tyr 383, Pro 496, and Thr 499, are stably maintained through NKCC1’s conformational transition (Figure S12C). The exception is the interaction between the bound K^+^ and residue Asn 298, which changes during the conformational transition. This K^+^ is initially tightly bound in the OO state (average distance to Asn 298 = 2.9 ± 0.3 Å) to become loosely bound in the Occ and IO states (average distance to Asn 298 = 4.1 ± 0.8 and 4.4 ± 0.7 Å, respectively). The interaction with Thr 499 also changes during the conformational transition, although this occurs at a different stage of the process. In both OO and Occ states, K^+^ remains tightly bound (average distance to Thr 499 = 2.7 ± 0.2 and 2.8 ± 0.2 Å, respectively) but becomes loosely bound in the IO state (average distance to Thr 499 = 4.3 ± 1.6 Å). On the other hand, the second Cl^–^ has a distinctive binding mode to its binding site (closest to the extracellular side), which is composed by residues: Val 302 and Met 303. In the OO state, it is loosely bound to both Val 302 and Met 303 (average distance to each residue being 4.5 ± 1.7 and 5.2 ± 1.8 Å, respectively). Whereas it is tightly bound in the occluded state (average distance to each residue being 2.4 ± 0.2 and 2.6 ± 0.2 Å, respectively) and IO state (average distance to each residue being 2.5 ± 0.3 and 2.7 ± 0.3 Å, respectively). These results further support that the second Cl^–^ has the propensity of spontaneously binding to the OO state.

### NKCC1 Permeability Allows Water Transportation

**Figure 10:**
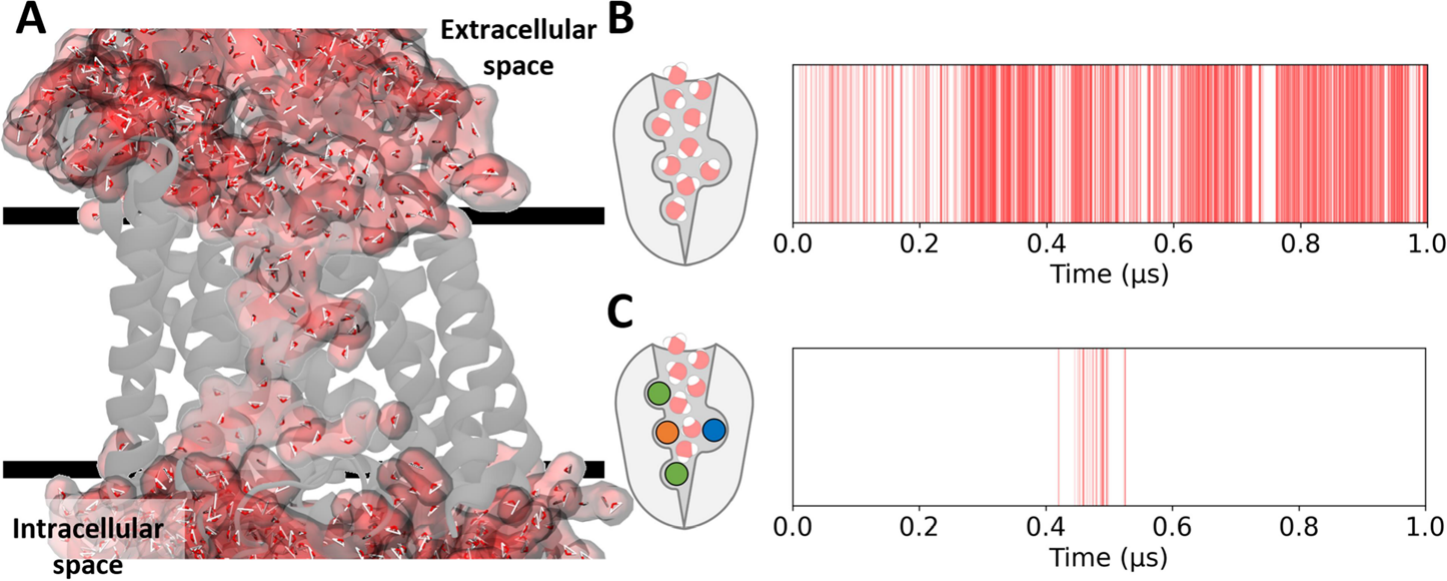
NKCC1’s outward open conformations are permeable to water. (A) Representation of NKCC1 (gray cartoon) embedded in the membrane (black horizontal bars) in a water-permeable state. Water molecules are shown as red and white lines with a red atomic surface representation. A chain of water molecules whose oxygen atoms are within 4.0 Å of each other connecting the extracellular and intracellular solvent is present. (B, C) Schematic representation of both states that present permeability to water (outward open without ions bound, B, or fully loaded, C), and their respective plot, which tracks the appearance of permeable states during each state’s equilibrium MD simulation. Vertical red bars represent snapshots from the respective simulation where a chain of water molecules whose oxygen atoms are within 4.0 Å of each other connecting the extracellular and intracellular solvent through NKCC1 is observed.

To better understand the capabilities to transport water of NKCC1, we modeled two new additional conformational states. These were IO and OO, which we loaded with two Cl^–^, Na^+^ ions, and K^+^ ions in their respective binding sites. These two fully loaded models allowed us to evaluate water permeability and water transport in NKCC1 in the presence of ions (as opposed to the previous models that were studied in the absence of ions). Notably, we observed an average of 13 water molecules trapped in the ion binding cavity through 100 ns of equilibrium MD simulations of the occluded state without ions bound. This value would then represent an estimation of the maximum amount of water molecules transported per alternating access cycle. Interestingly, our simulations also showed that NKCC1 adopts water-permeable states in the OO state (Figure 10A), preferentially when unloaded (38.2% of the trajectory, Figure 10B), while it does so only to a minor extent in the OO state with 4 ions bound (1.1%, Figure 10C). Finally, NKCC1 is not permeable at all in the loaded and unloaded IO and Occluded states.

**Figure 11:**
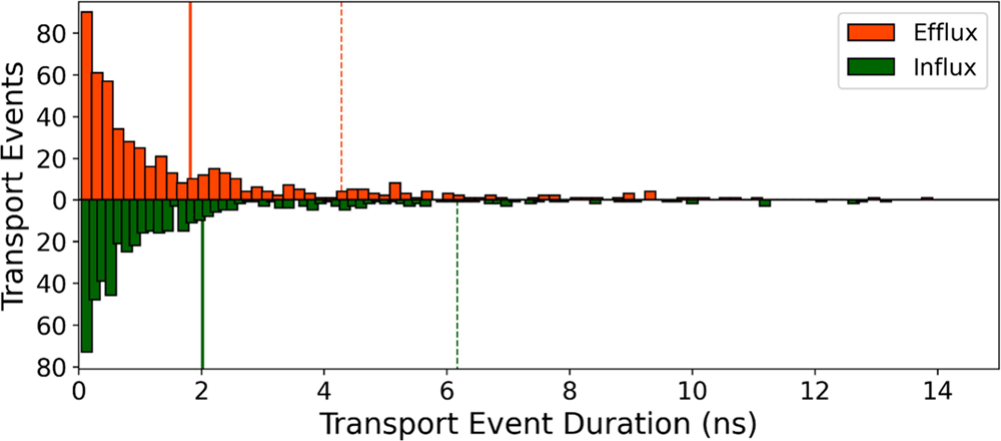
NKCC1 passively transports water. Histogram of all transport events detected in the state equilibrium MD simulation of NKCC1 outward open conformation with no ions bound, organized by the length of each transport event. Bars show the frequency of efflux/influx (orange/green) events per transport event duration. Vertical continuous lines show the mean, and discontinuous lines show the standard deviation (efflux, orange; influx, green). Some longer transport events were excluded from the histogram for clarity.

Water-permeable states of NKCC1 have an average lifetime of 0.5 ± 0.5 ns, with a median lifetime of 0.2 ns and a maximum lifetime of 5.0 ns in the unloaded OO conformation. On the other hand, water-permeable states in the OO fully loaded state have an average lifetime of 0.3 ± 0.2 ns, with a median of 0.2 ns and a maximum lifetime of 1.0 ns. This finding is in line with our mechanistic insights provided by the enhanced sampling simulations, which showed that in the IO states TM 10 modulates the hydration of the outer vestibule. Ultimately, when NKCC1 adopts a permeable state, water molecules diffuse across the membrane. During the 1 μs-long MD simulations of the OO state, we tracked 517 complete water molecule efflux events and 497 influx events (Figure 11). It is to be noted that these were pure diffusion events, as our MD simulations did not include any concentration gradient across the membrane. With this premise, we estimated the average water diffusion rate as 1.8 ± 2.5 and 2.0 ± 4.2 ns, in the outward and inward direction, respectively.

## Discussion

The CCC NKCC1 plays a crucial role in cellular osmolarity by regulating ionic balance and water flux,^29^ and it is currently targeted to treat a variety of related imbalance diseases, e.g. brain disorders including neurodevelopmental, neurodegenerative, neurological, disorders, hydrocephalus, as well as cancer.^3−7^ Motivated by the recent structural data on NKCC1 in different conformations—IO and OO states—we built and simulated atomistic models of *human* NKCC1, where the transporter is embedded in the membrane. We used these models to run multiple μs-long MD equilibrium and enhanced sampling simulations of those key IO and OO states and capture the exact protein dynamics for IO ↔ OO transitions.

Notably, our investigation led to a grand total of 16 conformational transitions observed using the OPES algorithm for enhanced sampling simulations.^19^ These simulations revealed a rocking-bundle mechanism for conformational transitions for the unloaded NKCC1 protein. Specifically, such a rocking-bundle mechanism shows alternate access to the NKCC1 ion binding sites through two concerted motions. First, helices TM 4 and TM 9, associated with each other by hydrophobic interactions, go through an angular motion of ∼14° between the IO and OO states. This is then coupled by the motion of TM 10, linked by a short extracellular loop to TM 9. The latter alternatively affects the bending or straightening of TM 10, thus blocking/allowing solvent access to the ion binding sites from the extracellular side. Such motions were consistently observed in all our IO ↔ OO state transitions (see Movie S1). We also observed these motions in our fully loaded NKCC1 model, suggesting that NKCC1 undergoes a rocking-bundle mechanism with and without bound ions. Remarkably, NKCC1’s TM 4, TM 9, and TM 10 behaviors are conserved in the LeuT-fold sodium-benzylhydantoin transporter Mhp1 from *Mycobacterium liquefaciens*,^30^ where the same TM helices may therefore carry out an analogous role for function.

Importantly, from our enhanced sampling simulations, we also identified the so-called “occluded state” for NKCC1, where the ion binding sites are inaccessible from both sides of the transporter (i.e., both the inner and outer vestibules are closed). Notably, the occluded state was only hypothesized to exist based on previous structural studies,^27^ although it has never been experimentally observed in any of the CCCs. On the other hand, in our simulations, we like to emphasize that the deep-learning-guided sampling of NKCC1 conformations detected such an occluded state without any previous knowledge of its existence. In other words, this previously uncharacterized occluded conformational state was not used to generate our data set and identify proper CVs for deep-learning enhanced sampling simulations, which therefore have unexpectedly revealed such a minimum on the FES, with no external solicitation. In addition, the OO state shows two distinct conformations, distributed into a shallow area located at ∼2.6 in the FES. These conformations (OO^w^ and OO^d^ in Figure 8) show a difference in the extent of their water hydration (Figure S7B) at the vestibules due to the modulatory motion of TM 10. Interestingly, the hydration level of the outer vestibule in the OO state was significantly affected by the Arg 307–Glu 389 salt bridge (Figure S8). It is also worth noting that the drug bumetanide is bound to the outer vestibule in all the available cryo-EM structures of NKCC1 in the OO state.^12,15^ Ligand binding at the outer vestibule hampers the formation of the Arg 307-Glu 389 salt bridge, which is, therefore, proven to be crucial for the rocking-bundle mechanism in NKCC1.

Overall, the IO state is found to be the lowest energy basin of the FES. From the IO state, the transporter transits to the OO state, passing through the occluded state. Our semiquantitative estimation of the energetic cost for the IO → OO transition is ∼10.5 kcal/mol, whereas the estimated cost for the OO → IO transition is ∼3.5 kcal/mol. Interestingly, these energetic estimates would also explain why all of the so-far resolved NKCC1 apo structures have been captured in the lowest free energy IO state minimum.

Another interesting observation is related to the possibility that NKCC1 can transport water molecules. Here, we have observed and quantified the ability of water to passively permeate through NKCC1. Using additional simulations with 4 ions bound to the IO and OO states, we could compare the presence of water molecules in different models and observe that NKCC1 indeed adopts water-permeable states exclusively in the unloaded OO state (38.2% of the trajectory, Figure 10B). We found that NKCC1’s water-permeable states have an average lifetime of 0.5 ± 0.5 ns, with water molecules that can diffuse across the membrane. In our MD simulations of the OO state, we tracked 517 complete efflux events and 497 influx events (Figure 11), which occur through diffusion (see Movie S2), as our MD simulations do not imply concentration gradient across the membrane. With this premise, we estimated the average water diffusion rate, which is 1.8 ± 2.5 and 2.0 ± 4.2 ns, in the outward and inward direction, respectively. Water transport may also take place via the alternating access cycle. During the simulation of the occluded state without bound ions, we observed about 13 water molecules trapped in the ion binding region. The latter can be therefore considered an estimation of the maximum amount of water molecules transported per alternating access cycle. Interestingly, these semiquantitative observation enrich the current experimental evidence for water transport by NKCC1^31,32^ and its functional implications in the context of CSF accumulation recently demonstrated.^33,34^ Indeed, water may cross the membrane via the formation of permeable states, as has been reported for a wide range of transporters, including Na^+^/glucose transporter (SGLT), glutamate transporter (Glt_ph_), glycerol-3-phosphate transporter (GlpT), sodium-benzylhydantoin transporter (Mhp1), and the maltose transporter,^35^ and more recently also shown for the sodium/proton antiporter PaNhaP.^36^

One more mechanistic feature from our simulations is the interhelical interface intrinsically associated with NKCC1’s transport capabilities. This interface is formed by the extracellular tip of helices TM 10 and TM 6. In particular, the interatomic contacts at the TM 10–TM 6 interface are maintained in the IO state (Figure 5), allowing sealing of the outer vestibule in the IO state. This interaction will then be broken to transit to the OO state, aided by the specific rocking-bundle mechanism and the associated helice motions (TM 4 and TM 9, Figure 2). To prove the relevance of such a critical protein interface, we also mutated a few residues located in it. From *mouse* NKCC1, we inserted the following mutations: Ala490Trp in TM 6, and Leu664Ala, Asn665Ala, and Ala668Trp in TM 10 (equivalent to *human* NKCC1 Ala497Trp, Leu671Ala, Asn672Ala, and Ala675Trp, respectively). All of these mutations led to a significant reduction in ion transport (Figure 6). This is also in line with previous cross-linking experiments, which highlighted the significant movement of TM 10 during NKCC1 ion transport.^37^ This further validates the key modulatory motions reported here for TM 10. Notably, TM 4’s functional importance is supported by mutational data showing that the Arg410Gln mutation—located at the extracellular extreme of TM 4—leads to a loss of function.^38^ Our computational evidence is therefore well supported by our and literature’s mutagenesis data.

Additionally, we characterized the energy profile of the binding of ions to the OO state of NKCC1. Crucially, we found that the Na^+^ dissociation energy in the OO state is 12.5 kcal/mol, while its dissociation energy in the IO state has been previously reported to be 6.9 ± 0.8 kcal/mol.^39^ We note that the free energy calculations for ion release that pertain to the IO state^39^ were performed via well-tempered metadynamics, while ours pertain to the OO state for ion binding and unbinding, computed using OPES Explore simulations. While qualitative, these results hint at anyhow to a higher binding affinity of Na^+^ to the OO state than to the IO state. Notably, this is congruent with NKCC1’s use of a Na^+^ electrochemical gradient to import Cl^–^ and its overall functional cycle—where ions bind to the OO state from the extracellular side of the membrane and are then released from the IO state into the intracellular side of the membrane.

### Extension of Our Mechanistic Implications to Na^+^-Dependent CCCs

Intriguingly, the results on the functional relevance of TM 4 and TM 9, along with TM 6 and TM 10, motivated our analysis of additional mutations of CCCs in this region, often linked to the insurgence of pathologies.^40^ In doing so, we found a total of 38 mutations (including the new 4 mutations reported here), which are all located on the functionally relevant helices identified in our work, namely, TM 4, TM 6, TM 9, and TM 10, highlighted in red in Figure S9. These four TM helices present an extremely high level of conservation (considering either identity and similarity) in Na^+^-dependent CCCs—between 80 and 100% when compared to *human* NKCC1, as shown in Figure S9. On the other hand, these functionally relevant TM helices present a much lower degree of conservation in Na^+^-independent CCCs, with values ranging from 40 to 73% when compared to *human* NKCC1. Therefore, the rocking-bundle mechanism seems to operate only for Na^+^-dependent CCCs, to which NKCC1 belongs. Na^+^-independent CCCs (KCCs) may operate through a different alternate access mechanism, where transitions seem driven by different TM helices, like TM 3 and TM 8 in the *human* KCC1.^41^ In particular, we could only find three mutations in our region of interest in Na^+^-independent CCCs that lead to transporter dysfunction, and all these are in TM 6. However, these three mutations are reported to have functional effects that seem unrelated to the rocking-bundle alternating access mechanism. Specifically, the *human* KCC2’s Leu426Pro mutation leads to complete loss of protein function, with reduced expression and glycosylation.^42^ The *human* KCC2’s Met438Val mutation alters the Cl2 binding site (referenced on the cited article as Met415Val because it is mutated in KCC2b) and therefore leads to impaired Cl^–^ extrusion.^42^ Finally, the Phe493CysfsX48 mutation in *human* KCC3 is associated with a frameshift mutation, which generally carries serious pathogenic consequences, like in this case with agenesis of the corpus callosum.^43^

## Conclusions

In summary, our computational simulations and free energy calculations, coupled to mutagenesis experiments, show that NKCC1 operates through the rocking-bundle mechanism, transiting from the unloaded IO to the OO states and vice versa. We also found that the OO states are permeable to water, which can freely go through NKCC1 across the membrane. Importantly, we found that most functionally relevant TM helices involved in such a mechanism are highly conserved in Na^+^-dependent CCCs. This overall evidence and critical mechanistic implications could open new strategies for NKCC1 function modulation and novel modes of drug action.

## Methods

### Equilibrium MD

The IO state model was constructed based on the cryo-EM structure of apo *human* NKCC1,^13^ using the TM domain of one monomer (comprising residues 288–753). The OO state model was built from the last snapshot from previous simulations,^15^ from which we removed bumetanide and bound ions and allowed water to flood the space previously occupied by the removed elements. For both states, the NKCC1 monomer was embedded in a POPC bilayer. PropKa 3.0^44^ was used to determine the ionization state of titratable residues, assuming pH 7—all calculated p*K*_a_ values were conducive to the standard ionization state at pH 7. The simulation box included NKCC1 accompanied by 221/308 POPC molecules, 59/63 Cl^–^, 29/31 Na^+^, and 31/33 K^+^ ions (in the bulk solution, corresponding to ∼150 mM concentration), and ∼19,300/22,000 water molecules. In total, ∼ 95,000/114,000 atoms and a simulation cell of 87 Å × 96 Å × 110 and 100 Å × 115 Å × 97 Å dimensions, for the IO and OO states, respectively. Initial configurations of each model were assembled using Packmol-Memgen,^45^ part of the AmberTools software package.

Equilibrium MD simulations were performed using the GPU version of the PMEMD code^46^ of the AMBER package.^47^ The protein was modeled using the ff14SB force field,^48^ Lipid17 for the POPC bilayer,^49^ TIP3P for water^50^ and for the ions.^51^ Periodic boundary conditions were employed, using the particle mesh Ewald method to calculate the long-range electrostatics.^52^ The real part of the electrostatic and van der Waals interactions were computed with a 10 Å cutoff. The SHAKE algorithm^53^ was used to constrain bonds involving hydrogen atoms, allowing an integration time step of 2 fs. Simulations were performed at a constant temperature (310 K) and pressure (1 bar). The POPC bilayer and water solvent were allowed to equilibrate around the protein during 200 ns of the MD simulation. After energy minimization, the system was gradually heated to 310 K, with the protein backbone atoms maintained close to their cryo-EM position by applying a harmonic restraint. Then, 1 μs of production equilibrium MD was performed for each IO and OO state NKCC1.

### Conformational CV Design

To guide the exploration of such complex systems and transitions, we applied the OPES Explore algorithm, which requires the use of effective CVs to accelerate a meaningful sampling of the free energy surface. By combining OPES Explore with the DeepLDA CV that we developed, our simulations could capture the complex dynamical transitions between open conformations of the inner and outer vestibules of the transporter. Using the IO and OO state equilibrium MD simulations, we selected all Cα–Cα pairs from all 12 TM helices, totaling to ∼75,000 Cα–Cα distances. These were then subsequently filtered, eliminating intra-TM pairs and keeping pairs only from adjacent helices. Then, we kept Cα–Cα pairs whose average distance was deemed a contact (<10 Å) in the IO state and not a contact in the OO state (>10 Å) and vice versa. Finally, we retained Cα–Cα distances that were significantly different between the IO and OO states, by eliminating those whose average was within two standard deviations between states. This resulted in a carefully curated selection of 90 Cα–Cα distances (see also Figure S2). From each 1 μs-long equilibrium MD, 50,000 data points per distance and per state were then fed to DeepLDA.^18^ This produced a deep-learning CV where the IO state was defined as −2.6 and the OO state was defined as 2.6.

### Water CV Design

The conformational CV was sufficient for enhanced sampling simulations to transit between the IO and the OO states, but we observed that these simulations were getting trapped in a state with the outer vestibule open and highly solvated. To aid water flushing from the outer vestibule and reduce steric hindrance of water impeding structural rearrangements, we included a second water-focused CV.

This CV consisted of the coordination number between a virtual atom placed in the center of the outer vestibule (Figure S3) and the oxygen atoms of water molecules.^54,55^ The coordination was calculated using a switching function (see Formula 1) with the following parameters: *r*_0_ = 8.0 Å, *d*_0_ = 0, *n* = 2, and *m* = 8. This CV characterizes the water content of the outer vestibule, and when its fluctuations were enhanced through a bias potential, we were able to obtain 16 IO ↔ OO state transitions:
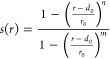
1

Switching function for the outer vestibule virtual atom–water coordination number.

### OPES Explore

We performed bidimensional OPES Explore^19^ on the DeepLDA CV and outer vestibule water coordination CV through PLUMED 2.8.^56^ We set an initial barrier value of 20 kcal/mol, a kernel deposition rate of 500 steps, and a 310 K temperature. The initial sigma was set to 0.1 and 0.3, and the minimum sigma was set to 0.05 and 0.15, for the conformational and water CV, respectively.

### Fully Loaded NKCC1 Conformations and Ion Binding Simulations

Fully loaded IO *human* NKCC1 was obtained by placing ions in their binding sites in accordance to their position in the zebrafish NKCC1 structure,^11^ except for the Na^+^ cation, which was placed in the center of mass of the coordinating atoms of the known Na2 binding site also identified in *human* NKCC1.^13^ We obtained a stable fully loaded IO NKCC1 conformation, in agreement with previous computational studies.^39^ This conformational state was then simulated for 1 μs.

Fully loaded occluded *human* NKCC1 was obtained by applying distance restrictions to each ion and all coordinating atoms, from each ion’s respective binding site. The application of these restraints, for 100 ns, led to a NKCC1 state with all four ions bound and inaccessible to the solvent from both extracellular and intracellular sides of the membrane—a fully loaded occluded state. After restrictions were lifted, this state was maintained for 120 ns of equilibrium MD, after which NKCC1 was transitioned into a fully loaded IO state.

Fully loaded OO *human* NKCC1 was obtained by starting from previous simulations,^15^ where NKCC1 was bound to bCl^–^ (Cl^–^ anion closest to the intracellular side), K^+^, and the inhibitor bumetanide. Bumetanide was removed, K^+^ was exchanged for Na^+^ from the solvent, and the outer vestibule was allowed to be filled with water. After equilibration, we ran OPES Explore simulation biasing two CVs. The first CV is the distance between the center of mass of the Na^+^ site coordinating atoms and the Na^+^ cation, currently bound to the K^+^ site. The second CV was the coordination number between the Na^+^ cation and the coordinating atoms of the Na^+^ site. We then selected a snapshot where the Na^+^ cation was within its binding site and ran for 100 ns of equilibrium MD. We observed that bCl^–^ and Na^+^ both stayed in their respective binding site through the 100 ns of simulation. We then selected the closest K^+^ to the outer vestibule from the solvent and ran a second OPES Explore over two similar CVs, but instead considering the K^+^ cation and the K^+^ binding-site coordinating atoms. Then, a snapshot where K^+^ was bound to its site was selected and used for an equilibrium MD. After an initial run of 100 ns, we observed that not only bCl^–^, Na^+^, and K^+^ remained in their respective binding sites but spontaneous binding of tCl^–^ to the top Cl^–^ binding site was observed very soon after the equilibrium MD started. Given that this last simulation was of NKCC1 in an OO fully loaded state, we extended it to 1 μs.

Na^+^ and Cl^–^ binding calculations were performed as described in the previous paragraph. OPES Explore simulations of Na^+^ binding started from a snapshot from our unloaded OO state NKCC1 equilibrium MD simulation. A snapshot where a Na^+^ ion was nearby the outer vestibule was selected and bias was applied to the distance between the ion and the center of mass of the coordinating atoms of the Na^+^ binding site and to the coordination number between Na^+^ and the coordinating atoms. OPES Explore simulations of Cl^–^ binding started from the previously equilibrated MD simulation of Na^+^/Cl^–^-bound OO state NKCC1, using the same set of CVs, considering Cl^–^ and its respective binding site. Ion binding order to the OO state of NKCC1 (Na^+^, Cl^–^, K^+^, and Cl^–^) was determined from experimental evidence.^28^

For the OPES Explore simulations of NKCC1 loading, the barrier was set to 5 kcal/mol, a kernel deposition rate of 500 steps, and a 310 K temperature. Both initial and minimum sigmas were set to adaptive for both CVs. The coordination for all ions was calculated using a switching function (see Formula 1) with the following parameters: *r*_0_ = 2.35 Å, *d*_0_ = 0, *n* = 2, and *m* = 8.

### Water Permeability and Transport

Water permeability was defined as the presence of a network of water molecules, connected by a distance of at most 4.00 Å between oxygen atoms from water molecules that encompassed the whole ion translocation pathway, connecting the extracellular and intracellular solvent (Figure 10).

To determine water transport in our simulations, we tracked each individual water molecule. An efflux event was determined to have happened when a water molecule crossed the inner membrane leaflet plane, the plane of the membrane bilayer, and then the outer membrane leaflet plane in this sequence specifically. An influx event was determined to have happened because a water molecule went through these planes in the reverse direction. Analysis was mostly performed with the MD analysis package.^57^

## Experimental Section

### Generation of NKCC1 Mutants and Cl^–^ Influx Assay

#### Generation of Mutants

Mutants of residues located in TM 6 and TM 10 in *mouse* NKCC1 [Ala490 (TM 6), Leu664 (TM 10), Asn665 (TM 10), Ala668 (TM 10), and mutating to Ala or Trp] were designed based on MD simulations. NKCC1 mutants (Ala490Trp, Leu664Ala, Asn665Ala, and Ala668Trp) were designed starting from the full-length mouse NKCC1-WT protein sequence cloned in the vector pRK5 (obtained from the Medical Research Council and the University of Dundee). The mutants were prepared by GenScript. For each mutant, the lyophilized DNA was resuspended and used to transform *Escherichia coli* TOP10 competent cells, and a maxi prep was performed to purify the DNA of each mutant. The sequences were then confirmed by Sanger sequencing.

#### Cl Influx Assay

HEK293F cells were cultured in Dulbecco’s modified Eagle's medium (DMEM) supplemented with 10% fetal bovine serum, 1% l-glutamine, 100 U/mL penicillin, and 100 μg/mL streptomycin and maintained at 37 °C in a 5% CO_2_ humidified atmosphere. To assess WT and mutant NKCC1 activity, 3 million HEK cells were plated in a 10 cm cell-culture dish and transfected with a transfection mixture comprising 5 mL of DMEM, 4 mL of Opti-MEM, 8 μg of DNA plasmid (pRK5 vector) coding for NKCC1-WT, NKCC1-A490W, NKCC1-L664A, NKCC1-N665A, NKCC1-A668W, or mock control (empty vector), together with 8 μg of a plasmid coding for the Cl-sensitive variant of the mbYFPQS (Addgene plasmid #80742), and 32 μL of Lipofectamin 2000. After 4 h, the cells were collected and plated in 96-well black-walled, clear-bottomed plates at a density of 250,000 cells/well. After 48 h, cells were used for the Cl influx assay. All reagents were purchased from Life Technologies unless otherwise specified. The Cl influx assay was performed in transfected cells treated with DMSO in 200 μL/well of a Cl-free-hypotonic solution (67.5 mM Na Gluconate, 2.5 mM K Gluconate, 15 mM HEPES pH 7.4, 5 mM Glucose, 1 mM Na_2_HPO_4_, 1 mM NaH_2_PO_4_, 1 mM MgSO_4_, and 1 mM CaSO_4_). After 30 min of incubation, plates were loaded into a multiplate reader (Tecan Spark) equipped with an automatic liquid injector system, and fluorescence of Cl-sensitive mbYFPQS was recorded with excitation at 485 nm and emission at 535 nm. For each well, fluorescence was first recorded for 20 s of baseline and for 60 s after delivery of a NaCl-concentrated solution (74 mM final concentration in the assay well). Fluorescence of Cl-sensitive mbYFPQS is inversely correlated to the intracellular Cl^–^ concentration, therefore, Cl influx into the cells determined a decrease of mbYFPQS fluorescence. To quantify the average effects as represented by the bar plots, we expressed the decrease in fluorescence upon NaCl application as the average of the last 10 s of Δ*F*/*F*0 normalized traces. Moreover, for each experiment, to account for the contribution of Cl^–^ changes that were dependent on transporters/exchangers other than NKCC1, we subtracted the value of the last 10 s of Δ*F*/*F*0 normalized traces obtained from mock-transfected control cells from the respective Δ*F*/*F*0 values obtained from the cells transfected with WT or mutated NKCC1s. We then presented in the figure all the data as a percentage of the fluorescence decrease vs the value of the WT NKCC1-transfected cells.

## Data Availability

Trajectories are available upon request, while representative structures from all states (inward open, occluded and outward open in both fully loaded and ion-free states) are available in MJRM’s github repository (https://github.com/themanuelr/NKCC1_rep_struc).

## Supporting Information Available

Alternating access mechanisms, collective variable design, and relevant TM helices sequence alignment, and further analysis of the equilibrium and enhanced sampling MD simulations (state stability, salt-bridge formation and water content, and others) (PDF)
Angular motion for the rocking-bundle mechanism (MP4)
Presence of water-permeable states (MP4)
