# Supplementary material for: Cation Chloride Cotransporter NKCC1 Operates through a Rocking-Bundle Mechanism
Source: J Am Chem Soc. 2023 Dec 26;146(1):552–66. doi: 10.1021/jacs.3c10258 (PMC10786066; doi:10.1021/jacs.3c10258)
Supplement: Supplementary file 1 — ja3c10258_si_001.pdf [file ja3c10258_si_001.pdf]

SUPPORTING INFORMATION

The cation chloride cotransporter NKCC1 operates through a rocking-bundle mechanism

Manuel José Ruiz Munevar[1], Valerio Rizzi[2],Corinne Portioli[3,5], Pietro Vidossich[1],

Erhu Cao[6], Michele Parrinello[4,*], Laura Cancedda[5,*], Marco De Vivo[1,*]

[1]Laboratory of Molecular Modelling & Drug Discovery, Istituto Italiano di Tecnologia, Genoa, Via Morego 30, 16163, Italy

[2]Biomolecular & Pharmaceutical Modelling Group, Université de Genève, Geneva, Rue Michel-Servet 1, CH-1211 4, Switzerland

[3]Laboratory of Nanotechnology for Precision Medicine, Istituto Italiano di Tecnologia, Genoa, Via Morego 30, 16163, Italy

[4]Laboratory of Atomistic Simulations, Istituto Italiano di Tecnologia, Genoa, Via Morego 30, 16163, Italy

[5]Laboratory of Brain Development and Disease, Istituto Italiano di Tecnologia, Genoa, Via Morego 30, 16163, Italy

[6]Department of Biochemistry, University of Utah School of Medicine, Salt Lake City, UT 84112-5650, USA. 2

*Corresponding authors

Emails:

michele.parrinello@iit.it

laura.cancedda@iit.it

marco.devivo@iit.it

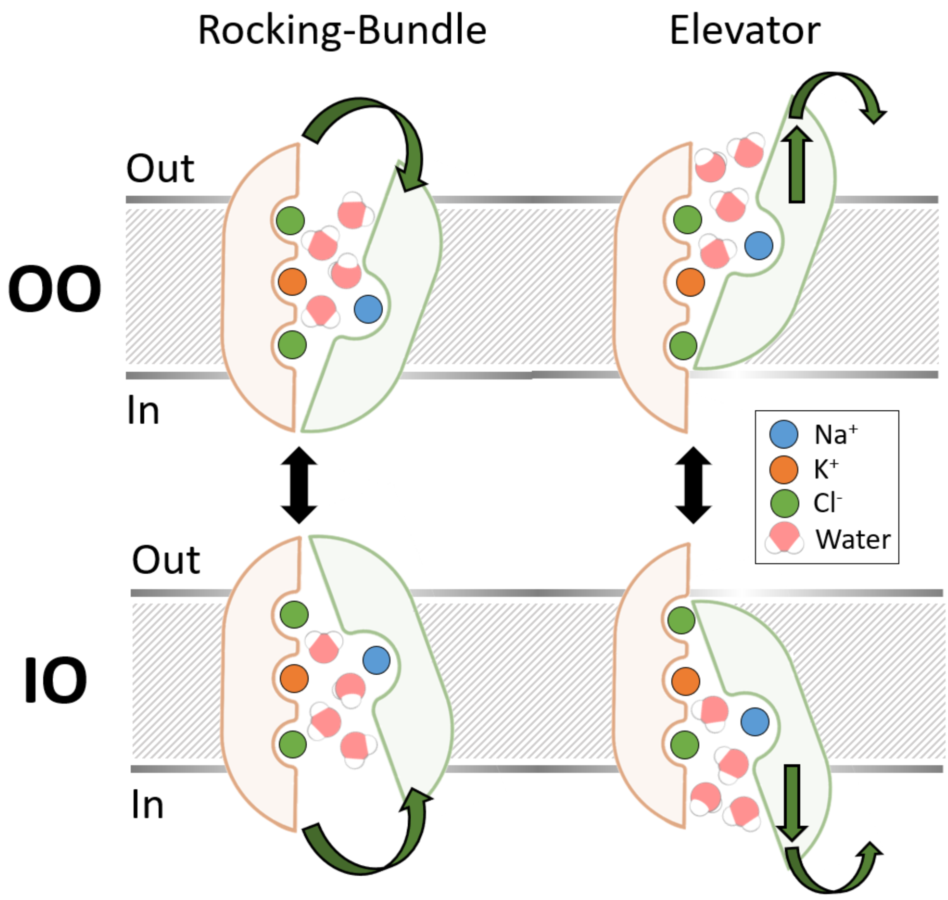

Figure SI 1: Different types of Alternating Access Mechanisms previously identified for the LeuT-fold transporters suggest possible conformational transition routes in NKCC1. On the left, the Rocking-Bundle Mechanism, where the mobile domain (light green) carries out an angular motion that alternates binding site accessibility to either side of the membrane. On the right, the Elevator Mechanism, where the mobile domain (light green) goes through vertical translation and an angular motion, to carry out alternation of binding-site accessibility.

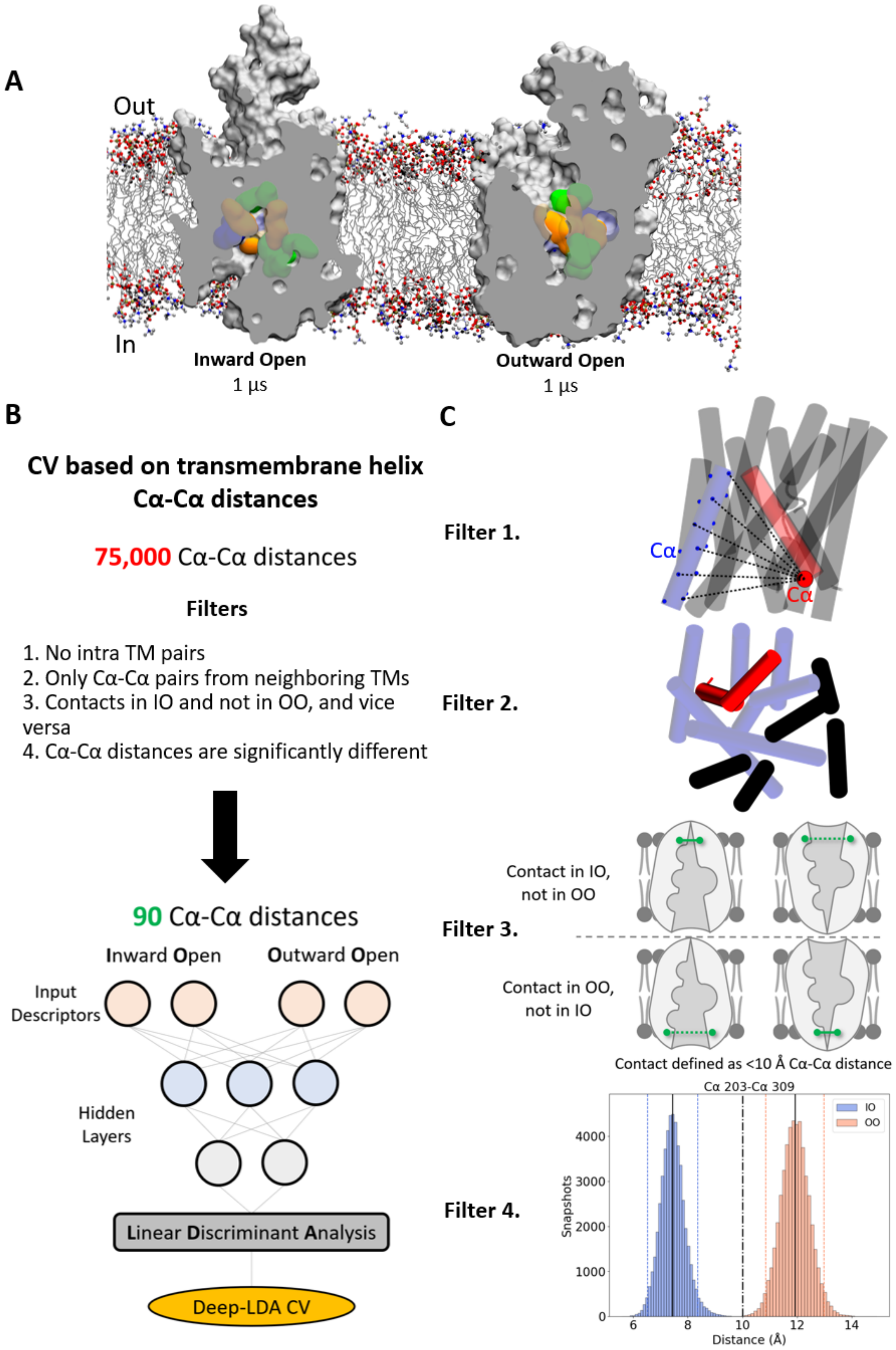
A
Out
In
Inward Open
1 μs
Outward Open
1 μs
B
CV based on transmembrane helix Cα-Cα distances
75,000 Cα-Cα distances
Filters
1. No intra TM pairs
2. Only Cα-Cα pairs from neighboring TMs
3. Contacts in IO and not in OO, and vice versa
4. Cα-Cα distances are significantly different
90 Cα-Cα distances
Inward Open
Outward Open
Input Descriptors
Hidden Layers
Linear Discriminant Analysis
Deep-LDA CV
C
Filter 1.
Cα
Cα
Filter 2.
Filter 3.
Contact in IO, not in OO
Contact in OO, not in IO
Contact defined as <10 Å Cα-Cα distance
Filter 4.
Cα 203-Cα 309
IO
OO
Snapshots
Distance (Å)

Figure SI 2: Collective variable design based on Deep Learning algorithms, which was essential for the elucidation of mechanism of NKCC1's conformational transitions. To explore the conformational transition between NKCC1's Inward Open and Outward Open states we looked for a collective variable with as little user input as possible. We used the Cα-Cα distances. We filtered and selected the Cα-Cα distances that had the capacity to pave the way for conformational exploration through OPES Explore. Our dataset was based on two independent equilibrium MD simulations of human NKCC1: 50,000 snapshots from 1 µs of Inward Open state, and other 50,000 snapshots from 1 µs of Outward Open state – (A) representation of both NKCC1 IO and OO states embedded in a membrane, coming from both our equilibrium MD simulations. (B) Diagram of the workflow to design the CV: From both states, we calculated all possible Cα-Cα distances from the 12 TM helices – in total ~75,000 pairs. We excluded from this initial step Cα-Cα pairs belonging to the same helix (e.g. residue 5 and residue 7 belong to TM 1, therefore their respective Cα was not calculated – C, Filter 1), and we excluded all pairs from non-neighboring helices (e.g. residue 5 and residue 55 belong to TM 4 and TM 11, who are too far apart to ever create or break bonds between them – C, Filter 2). We calculated the average distance for each Cα-Cα distance, and then kept for further analysis those whose average distance was defined as a contact (<10 Å) in one state, and not a contact (>10 Å) in the opposite state (C, Filter 3). Then, we kept only those Cα-Cα distances whose distribution was significantly different between the IO and OO state (C, Filter 4). This set of filters left us with 90 Cα-Cα distances that defined the conformational space between the NKCC1 IO and OO state. In this way, our dataset was constituted of 90 Cα-Cα distances over 100,000 equilibrium MD snapshots (50,000 from the IO state, and 50,000 from the OO state). This dataset was then fed into DeepLDA, which then generated a unidimensional collective variable. This variable describes as -2.6 the Cα-Cα distance distribution of the IO state and 2.6 the Cα-Cα distance distribution of the OO state.

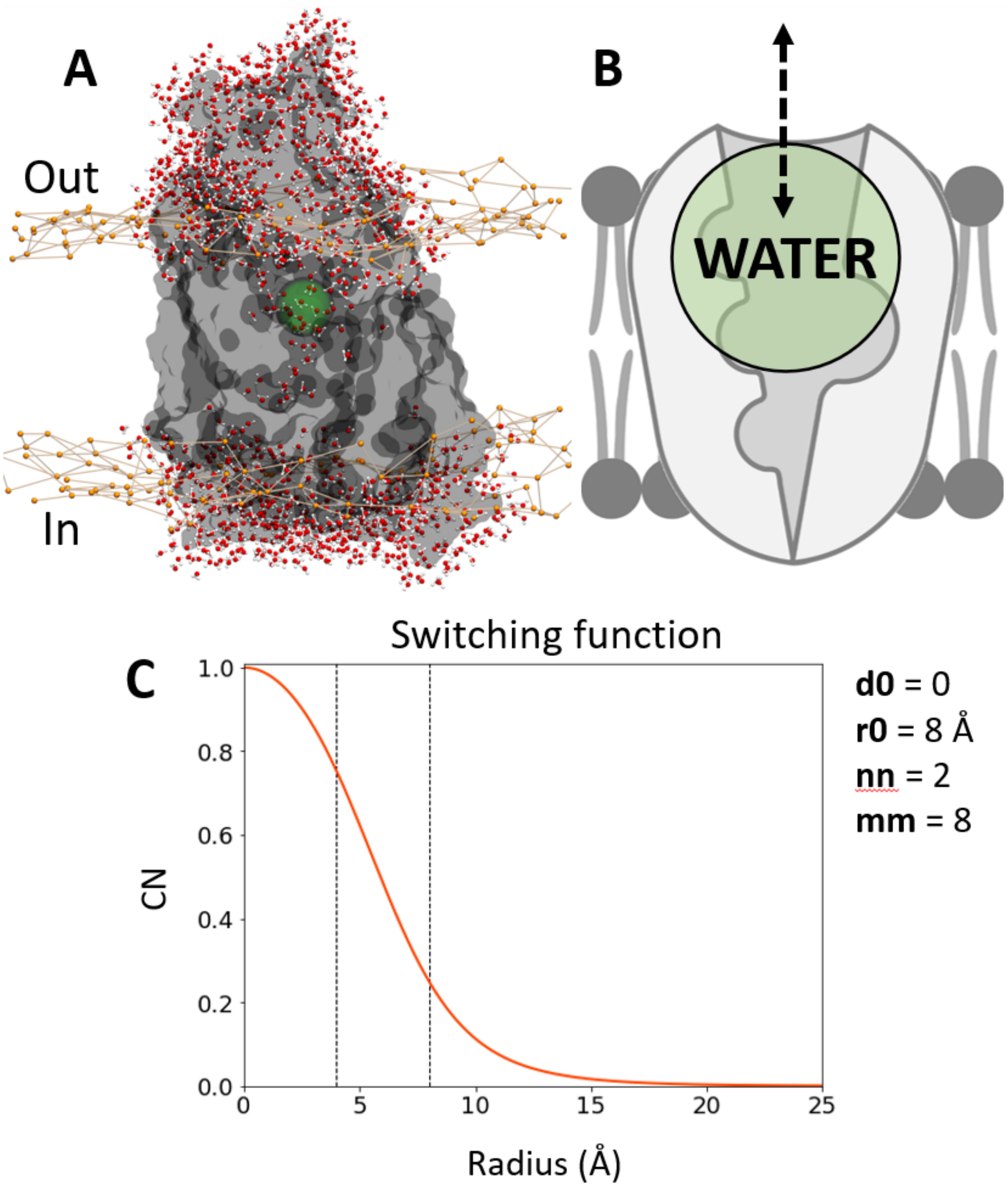

Figure SI 3: Collective variable design to account for water flow into the outer vestibule was necessary to efficiently sample the NKCC1 Inward Open ↔ Outward Open conformational transition. (A) Representation of human NKCC1, shown as a grey atomic surface, embedded in the cell membrane (orange wireframe with spheres), with a thick layer of water molecules (white sticks with a red sphere) around the protein and flooding the outer vestibule. The green sphere represents the location of where the virtual atom was positioned to calculate water oxygen coordination of the outer vestibule in all OPES Explore simulations. (B) Schematic representation of NKCC1 (light brown) embedded in the membrane, along with the virtual atom used to calculate water oxygen coordination (green circle). The dashed arrow showcases the direction of the water flow. (C) Plot of the switching function used to calculate water-oxygen coordination from the virtual atom placed in the outer vestibule. Dashed black lines highlight the coordination values of a water oxygen atom at 4 Å and 8 Å.

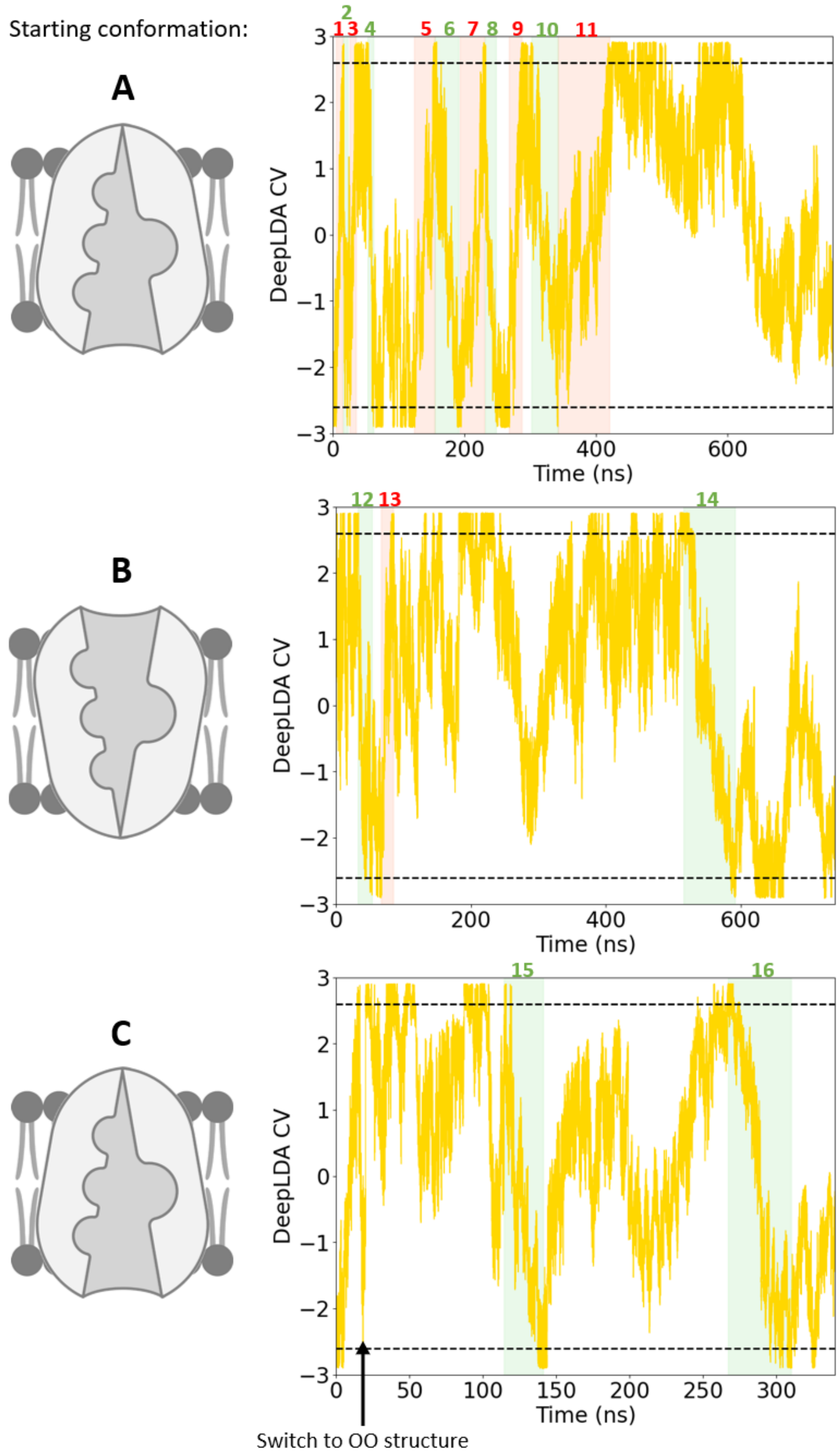

Figure SI 4: The DeepLDA CV and the coordination of water oxygens in the outer vestibule CV were succesfully used by OPES Explore to elucidate the Inward Open ↔ Outward Open NKCC1

conformational transition. (A-C) DeepLDA CV *vs* the simulation time of OPES Explore. IO → OO transitions are highlighted in red. OO → IO transitions are highlighted in green. These simulations started from the IO state (A) or the OO state (B), (C) corresponds to the plot of the first 20 ns of the IO starting simulations, whose structure was then manually exchanged to the OO structure. The dashed lines indicate -2.6 and 2.6, the values assigned by DeepLDA to the IO and OO state, respectively. The black arrow indicates when the structure was exchanged.

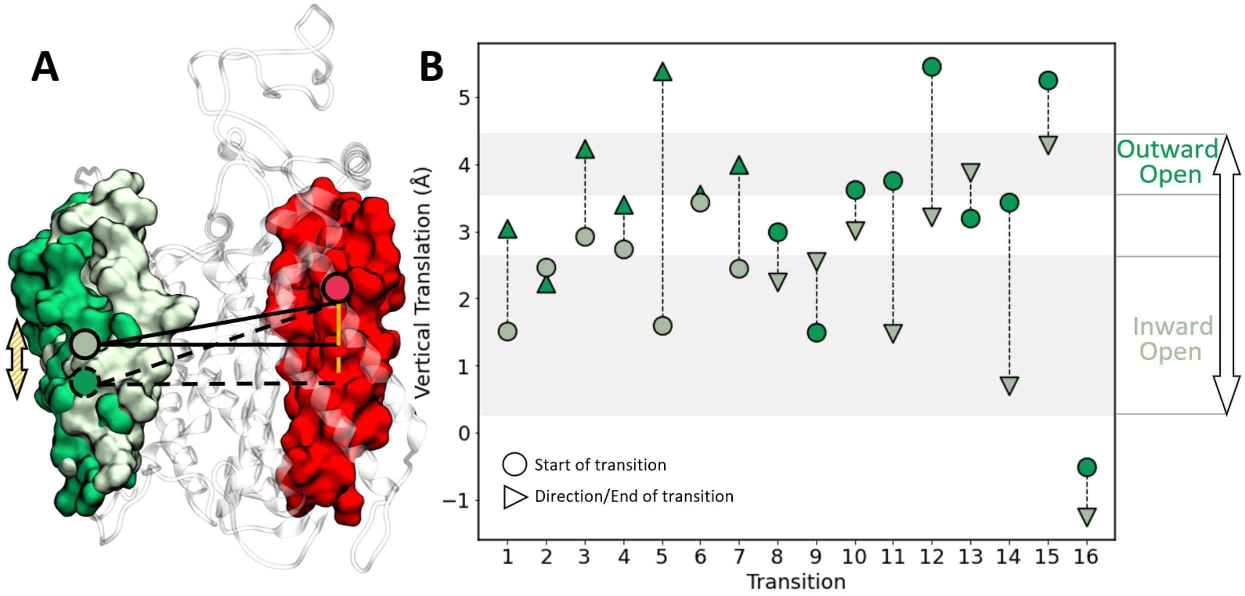

Figure SI 5: NKCC1 does not present vertical translation during its conformational transition between the Inward Open ↔ Outward Open conformations. (A) Schematic representation of NKCC1's vertical translation, defined as the difference between the Z coordinate of the center of mass of TM 4 and TM 9 (green surface for the TMs, green circles for the center of mass) *vs* the center of mass of TM 1, TM 2, TM 6 and TM 7 (the static domain, red surface for the TMs, red circle for the center of mass), between the OO and the IO state – bright and dim green, respectively. Black and dashed lines illustrate the relationship between centers of mass, yellow dashed line shows the quantified value in the Z coordinate. The striped arrow shows the expected vertical translation for the elevator mechanism. (B) Quantification of NKCC1's vertical translation of TM 4 and TM 9 with respect to TM 2 and TM 7 through 16 conformational transitions from OPES Explore simulations. The light brown horizontal bars represent the OO and IO average vertical translation ± 1SD from 1 µs of equilibrium molecular dynamic (MD) simulations. Circles represent the starting point of each transition, whereas the triangles represent the endpoint of the same transition and its direction. Circles and triangles are colored depending on the NKCC1 conformation they represent (bright green for OO and dim green for IO).

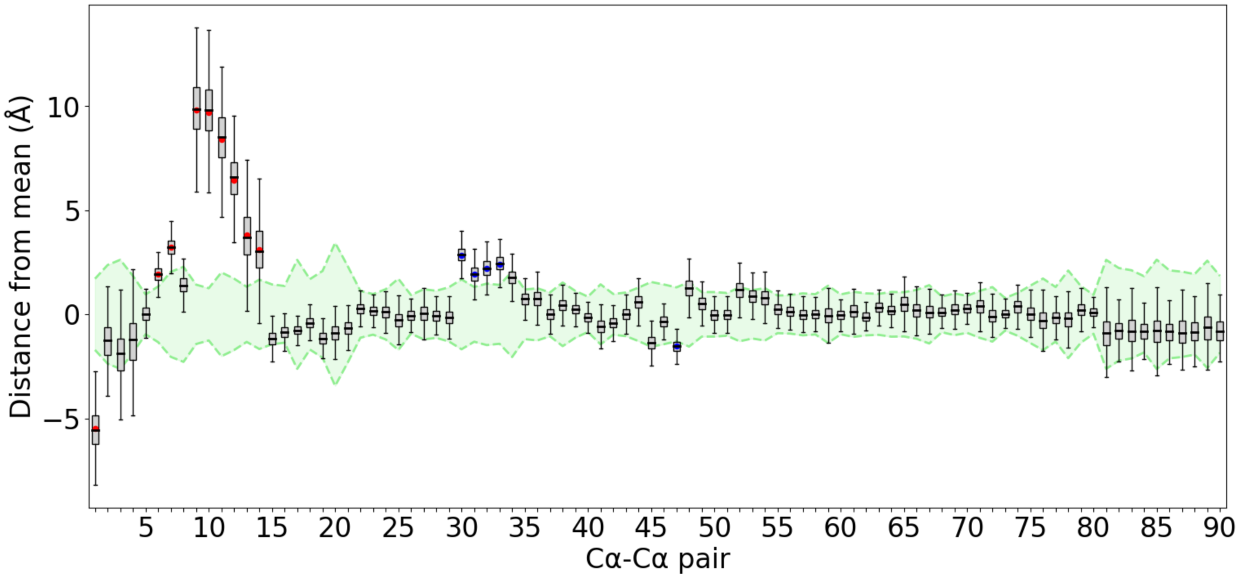

Figure SI 6: The shift from -2.6 of the DeepLDA CV value can be explained by the deviation of few Cα-Cα distances belonging to the first few residues of the NKCC1 model. Box plot distribution of the Cα -Cα pair distances from their respective MD mean in the IO state equilibrium. Those were calculated for all 90 distances that defined the DeepLDA CV. The 90 distances were measured from an equilibrium MD starting from a snapshot belonging to the IO basin in the FES. IO state equilibrium MD distance distribution is shown in green. Distances from residues belonging to the first bend of TM 1, far from their expected values, are highlighted in red. Distances highlighted in blue lay just outside their expected distribution, but did not lead to any structural determinant event.

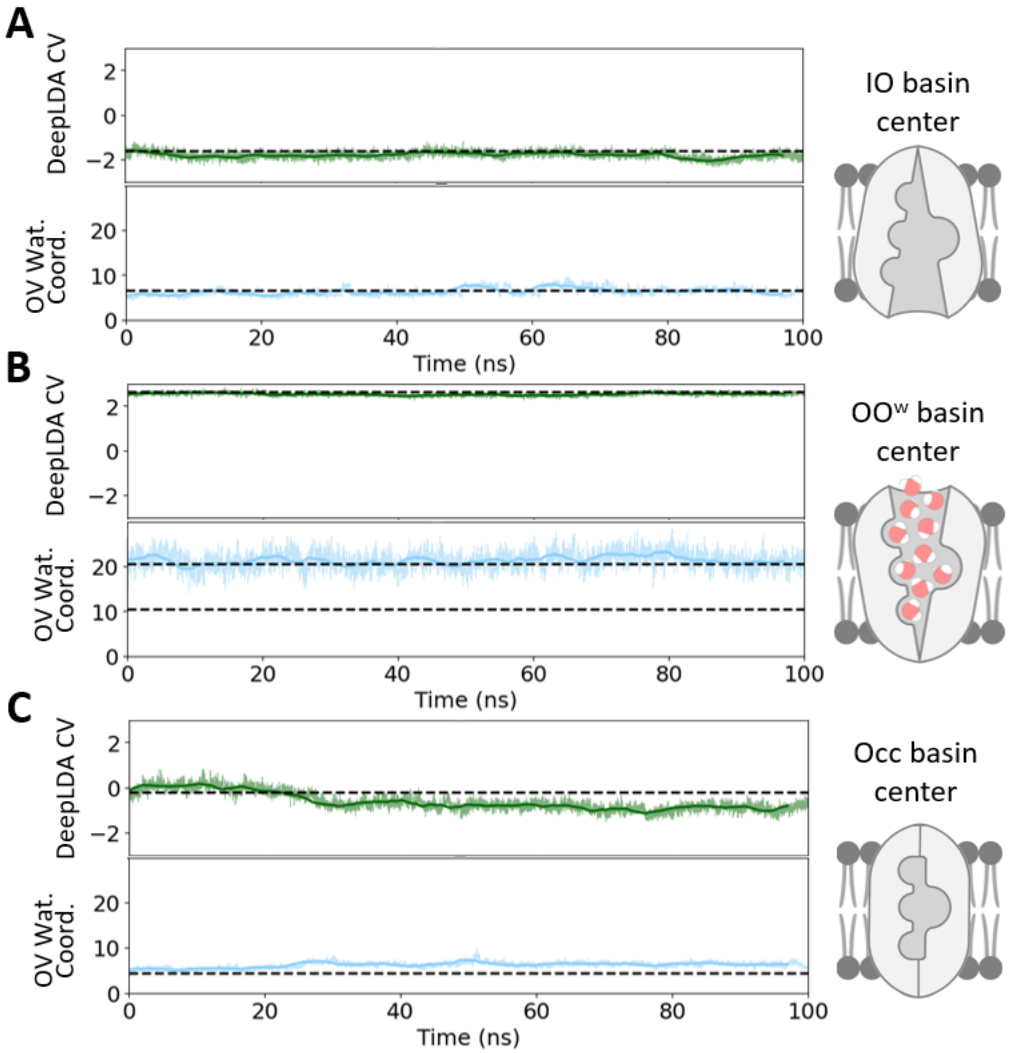

Figure SI 7: Equilibrium MD shows that snapshots from the FES's basins represent stable NKCC1 conformational states. (A-C) Plots of projections from equilibrium MDs of DeepLDA CV (top, green) and Water Coordination CV (bottom, blue). The equilibrium MD started from snapshots belonging to IO (A), OO^w (B) and Occ (C) basins in the FES shown in Figure 7. The respective schematic representation on the right shows NKCC1 conformations at the corresponding basins, represented by dotted lines in the plots.

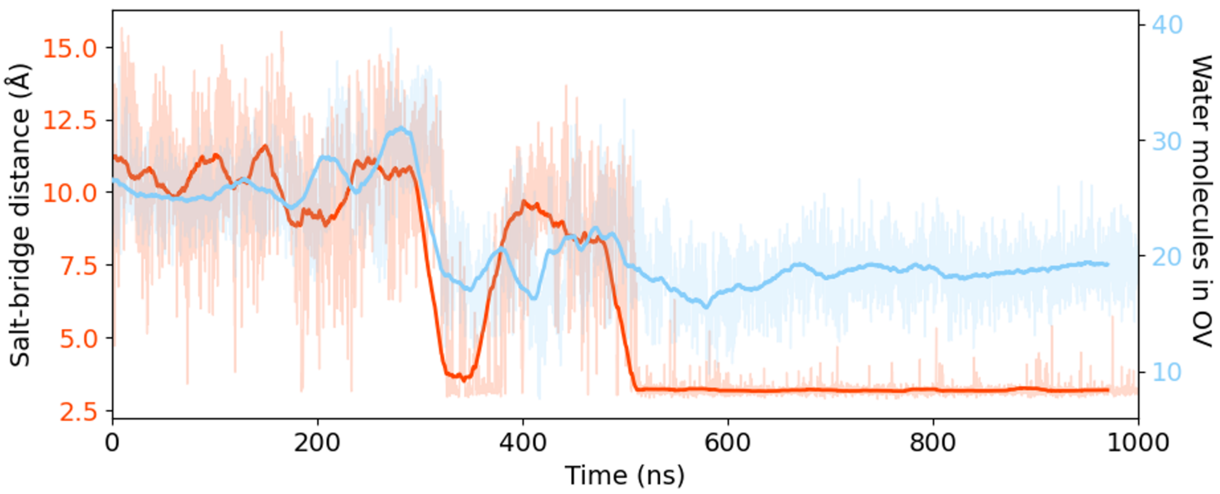

Figure SI 8: Decrease of outer vestibule hydration is correlated with the formation of a salt bridge in the Outward Open state equilibrium MD. Distance between charged atoms from the sidechains of salt-bridge forming residues Arg307 and Glu389 during the OO state equilibrium MD (orange), and the number of water molecules in the outer vestibule (OV, blue) during the same simulation.

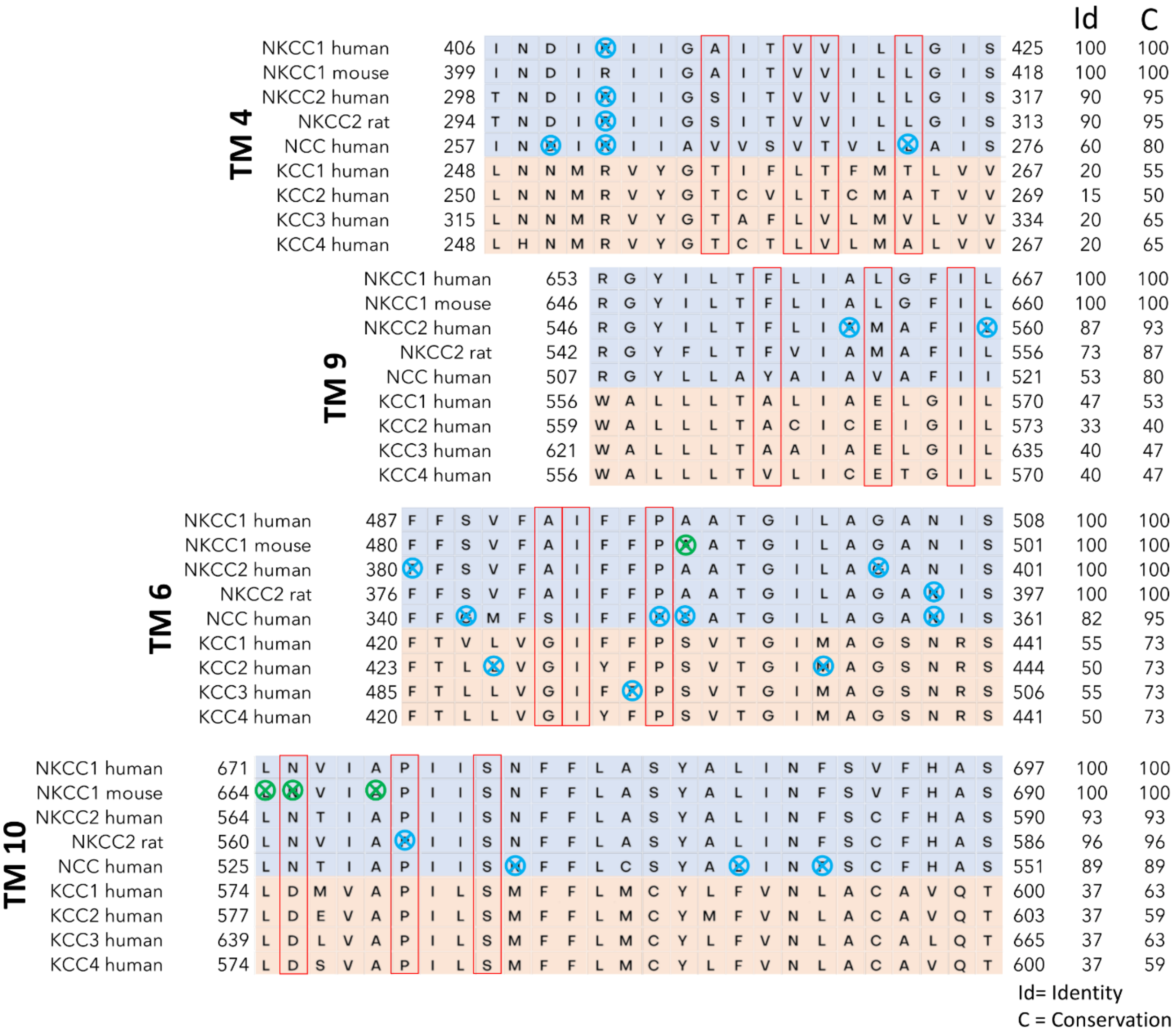

Figure SI 9: Alignment of NKCC1 with other CCC functionally relevant TMs show high conservation in Na$^+$-dependent transporters, low conservation in Na$^+$-independent transporters and prevalence of disease-associated mutation in only Na+ dependent transporters. Multiple sequence alignment of CCC's TM 4, TM 9, TM 6 and TM 10. Red rectangles highlight residues that participate in TM interactions relevant for our proposed rocking-bundle alternating access mechanism. Green circles highlight mutations used for our *in vitro* Cl$^-$ flux assay experiments performed on *mouse* NKCC1. Blue circles highlight mutations associated to human disease, as described in the literature (Portioli, et al., 2021).

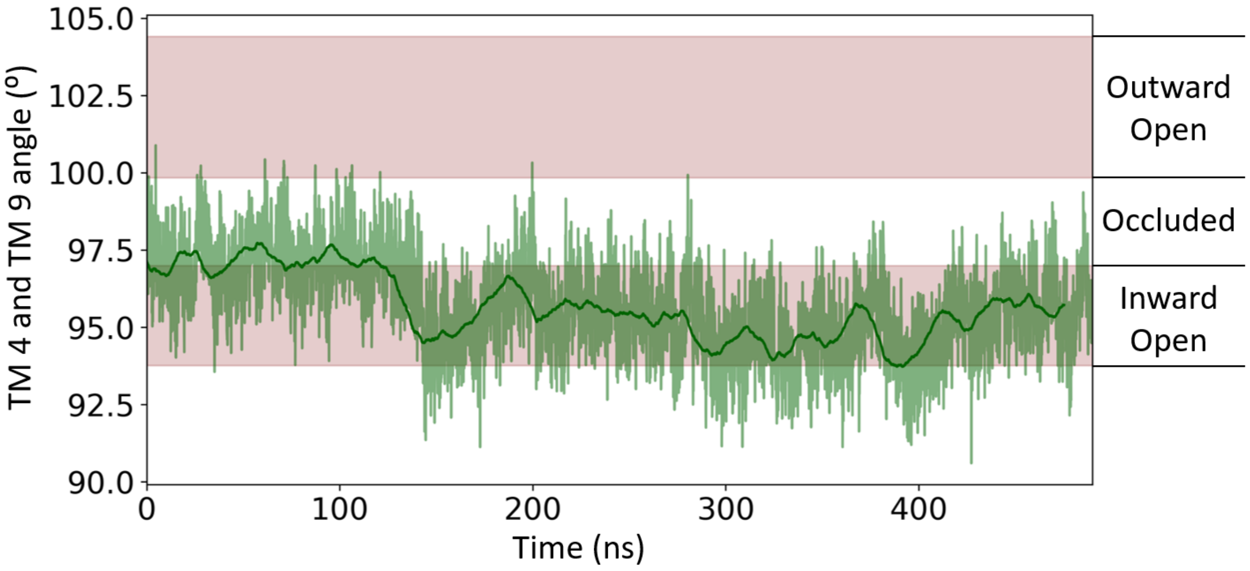

Figure SI 10: Fully loaded NKCC1 conformational transition undergoes the same molecular motions as unloaded NKCC1. Angular motion of TM 4 and TM 9 of fully loaded NKCC1 in the occluded conformational states. Equilibrium MD simulation of this state shows how the angular motions reflect an occluded state for the first 120ns, followed by a conformational transition into the fully loaded inward open state – where it stayed for the rest of the trajectory.

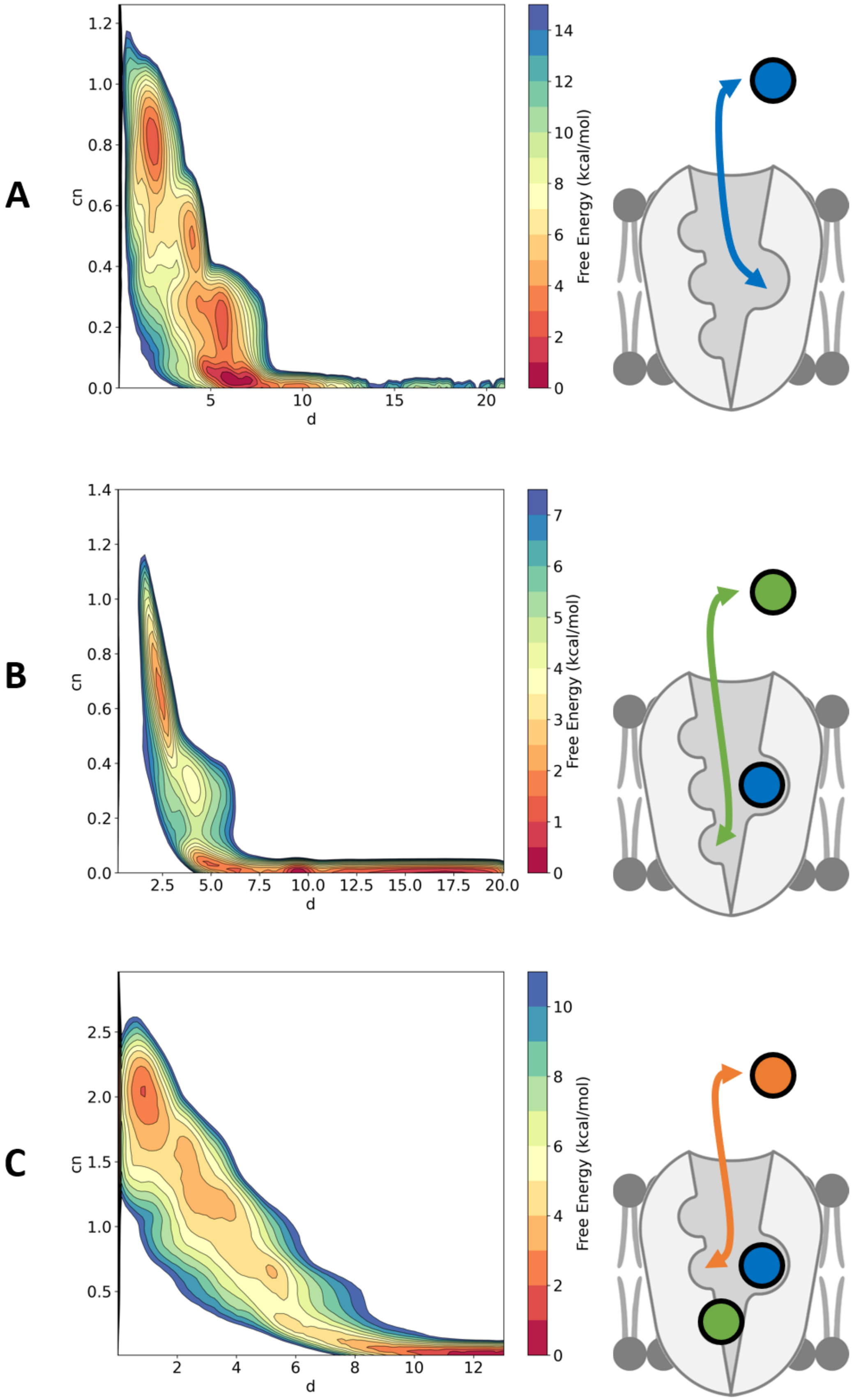
A
B
C
cn
d
Free Energy (kcal/mol)

Figure SI 11: Free Energy Surface of ion binding simulations. FES calculated by reweighting from OPES Explore simulations, biasing the distance between the relevant ion and the center of mass of the coordinating atoms and the coordination number of the coordinating atoms with respect to the ion. (A) FES for Na^+ binding in unloaded NKCC1. (B) FES for Cl^- binding in Na-loaded NKCC1. (C) FES for K^+ binding to Cl/Na-loaded NKCC1.

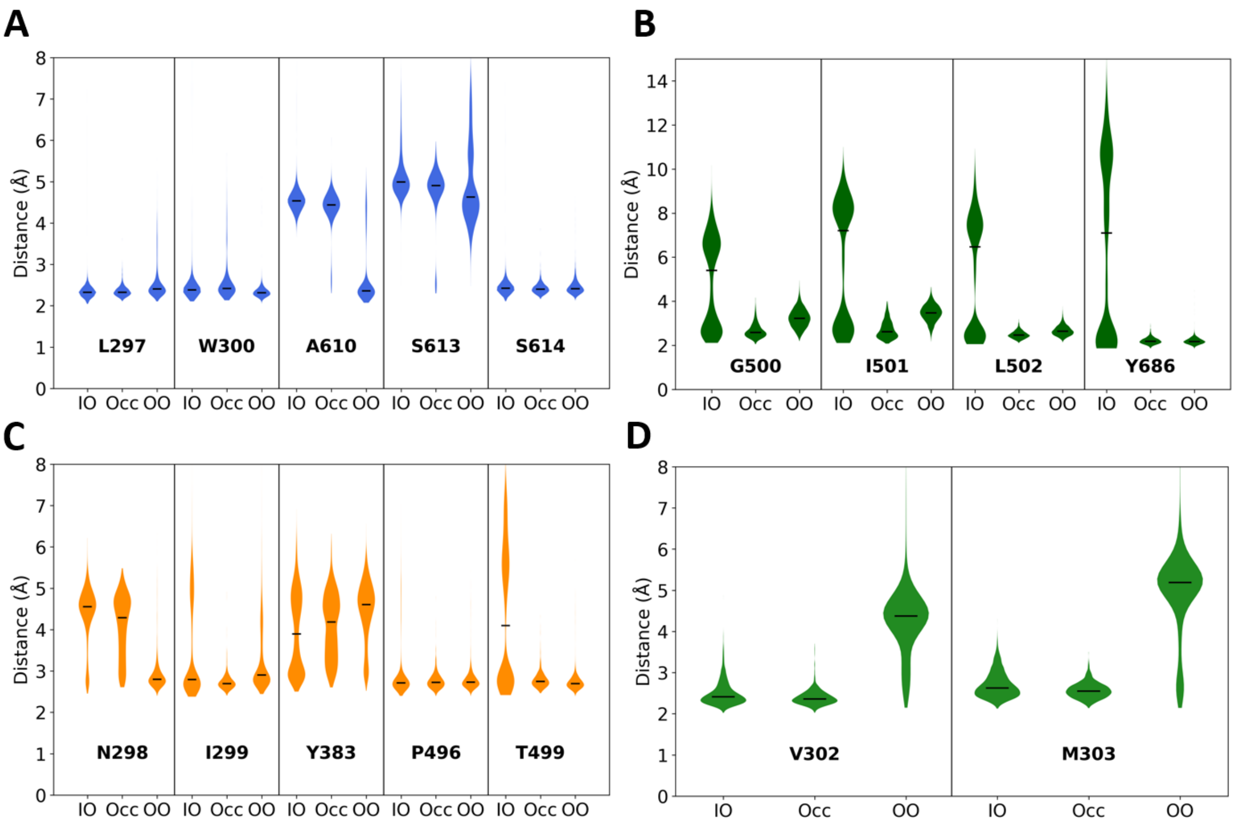

Figure SI 12: Binding mode of ions undergoes few changes during NKCC1's conformational transition. Violin plots represent the distribution of distances between the bound ion and the coordinating atoms from the respective binding site. (A) Distance distribution between Na^+ and its binding site, composed of oxygen atoms from residues: L297, W300, A610, S613, S614. In all fully loaded NKCC1 states, Na^+ remains tightly bound to L297, W300 and S614, and loosely bound to S613. On the other hand, interactions of Na^+ and A610 change during the conformational transition, starting tightly bound in the OO state, and loosely bound in both Occ and IO states. (B) Distance distribution between Cl^- and its binding site (closest to the intracellular side), composed by hydrogen atoms of residues: G500, I501, L502, Y686. bCl- remains tightly bound in all NKCC1 fully loaded states. We note that the distance distribution in the IO state is bimodal, where the lower distance mode represents a tightly bound Cl^-, and the higher distance mode represents states where bCl^- visits a secondary binding site. This site is in the vicinity of the intracellular gate (as identified by Janoš & Magistrato, 2021) and likely represents a state pre-ion release. (C) Distance distribution between K^+ and its binding site, composed of oxygen atoms of residues: N298, I299, Y383, P496, T499. In our fully loaded simulations K^+ remains tightly bound to I299 and P496, and loosely bound to Y383. Interactions between the bound K^+ and N298 change during the conformational transition, starting tightly bound in the OO state and becoming loosely bound in the Occ and IO states. Interactions with T499 also change during the conformational transition, although this change occurs at a different stage. In both OO and Occ states, K^+ remains tightly bound, but becomes loosely bound in the IO state. (D) Distance distribution between Cl^- and its binding site (closest to the extracellular side), composed by hydrogen atoms of residues: V302 and M303. Cl^- remains tightly bound to both residues in the Occ and IO state, and loosely bound to both residues in the OO state. This is consistent with the fact that Cl^-

spontaneously binds and unbinds in the equilibrium MD of the OO state – but then having its interactions stabilized as NKCC1's conformationally transitions into the Occ state.

Movie captions

Movie 1: NKCC1 conformational transition rocking-bundle mechanism. First, a scene showcasing the full simulated system, including water molecules as sticks (red and white), ions in solution as spheres (green – Cl^-, orange – K^+, blue – Na^+), the membrane bilayer as sticks (blue, red, and ochre sticks represent the head groups; grey sticks represent the hydrophobic tails), and the embedded NKCC1 as a yellow cartoon. Then, water is removed from the visualization for clarity. Second, we zoom into TM 4 and TM 9, shown as yellow cartoons (rest of NKCC1 is shown in grey cartoon). We observe the angular motion that TM 4 and TM 9 undergo when NKCC1 goes through the Inward Open (IO) → Outward Open (OO) conformational transition. TM 4 and TM 9 position in the IO state are shown as red cartoons. Then, we observe the OO → IO conformational transition and TM 4 and TM 9's angular motion, where their position in the OO state is shown as a blue cartoon. Third, we zoom into TM 10, shown as a yellow cartoon. We observe TM 10's solvent access modulation to the outer vestibule of NKCC1 when it goes through the IO → OO conformational transition, and TM 10's position in the IO state is shown as red. Once this transition is completed, the now accessible outer vestibule leading to the ion translocation pathway is highlighted with a discontinuous green circle. We then observe the OO → IO conformational transition, where TM 10's position in the OO state is shown in blue cartoon.

Movie 2: NKCC1 water permeability and a water efflux event. First, a scene showcasing the full simulated system, including water molecules as sticks (red and white), ions in solution as spheres (green – Cl^-, orange – K^+, blue – Na^+), the membrane bilayer as sticks (blue, red, and ochre sticks represent the head groups; grey sticks represent the hydrophobic tails), and the embedded NKCC1 as a yellow cartoon. Then, water is removed from the visualization for clarity. Second, we show NKCC1 as a white cartoon along with the closest 1000 water molecules as sticks. Then, we add a red surface that defines the van der Waals surface of the water molecules and remove the protein cartoon representation. This is done to show how NKCC1 is in a water permeable state, where the solvent from the extracellular and intracellular side is connected by a chain of water molecules interacting with each other. Third, we show – again – NKCC1 as a white cartoon along with the closest 1000 water molecules as sticks. A single water molecule is highlighted as red and white spheres. We then observe how the highlighted water molecule goes from the intracellular to the extracellular side through NKCC1, in what we call an efflux event.

Acknowledgements

Figures 1, 2, 6, 8, 9, 10, and SI Figures 1, 2, 3, 4, and 7 contain elements created with BioRender.com.
